# Distribution and Diversity of Soil Microfauna from East Antarctica: Assessing the Link between Biotic and Abiotic Factors

**DOI:** 10.1371/journal.pone.0087529

**Published:** 2014-01-31

**Authors:** Alejandro Velasco-Castrillón, Mark B. Schultz, Federica Colombo, John A. E. Gibson, Kerrie A. Davies, Andrew D. Austin, Mark I. Stevens

**Affiliations:** 1 Australian Centre for Evolutionary Biology and Biodiversity, School of Earth and Environmental Sciences, The University of Adelaide, Adelaide, South Australia, Australia; 2 Department of Genetics, Bio21 Institute, The University of Melbourne, Parkville, Victoria, Australia; 3 Hawkesbury Institute for the Environment, University of Western Sydney, Richmond, New South Wales, Australia; 4 Institute of Marine and Antarctic Studies, University of Tasmania, Hobart, Tasmania, Australia; 5 Australian Centre for Evolutionary Biology and Biodiversity, School of Agriculture Food and Wine, The University of Adelaide, Urrbrae, Adelaide, South Australia, Australia; 6 South Australian Museum, Adelaide, South Australia, Australia; 7 School of Pharmacy and Medical Sciences, University of South Australia, Adelaide, South Australia, Australia; University of Maryland, United States of America

## Abstract

Terrestrial life in Antarctica has been described as some of the simplest on the planet, and mainly confined to soil microfaunal communities. Studies have suggested that the lack of diversity is due to extreme environmental conditions and thought to be driven by abiotic factors. In this study we investigated soil microfauna composition, abundance, and distribution in East Antarctica, and assessed correlations with soil geochemistry and environmental variables. We examined 109 soil samples from a wide range of ice-free habitats, spanning 2000 km from Framnes Mountains to Bailey Peninsula. Microfauna across all samples were patchily distributed, from complete absence of invertebrates to over 1600 specimens/gram of dry weight of soil (gdw), with highest microfauna abundance observed in samples with visible vegetation. Bdelloid rotifers were on average the most widespread found in 87% of sampled sites and the most abundant (44 specimens/gdw). Tardigrades occurred in 57% of the sampled sites with an abundance of 12 specimens/gdw. Nematodes occurred in 71% of samples with a total abundance of 3 specimens/gdw. Ciliates and mites were rarely found in soil samples, with an average abundance of 1.3 and 0.04 specimens/gdw, respectively. We found that microfaunal composition and abundance were mostly correlated with the soil geochemical parameters; phosphorus, NO_3_^−^ and salinity, and likely to be the result of soil properties and historic landscape formation and alteration, rather than the geographic region they were sampled from. Studies focusing on Antarctic biodiversity must take into account soil geochemical and environmental factors that influence population and species heterogeneity.

## Introduction

Desert ecosystems are often regarded as some of the simplest on Earth, in terms of trophic levels and biodiversity, which is contrasted to temperate and tropical ecosystems [1]. In hot desert environments, soil microfaunal composition and diversity are linked to plant distribution and organic matter accumulation [2]. Water has also been shown to be a potential determinant for species diversity in these kinds of environments [3], [4]. Examination of hot and cold deserts, often lacking vascular plants and where water is a limiting factor, offers the opportunity to understand biotic interactions at multiple spatial scales. Such interactions are difficult to elucidate in less extreme environments that tend to have more intricate soil structures [2], [5]. Organisms that survive in Antarctic (cold desert) refuges are constantly subjected to extreme abiotic stresses such as low temperatures, freeze-thaw cycles, available liquid water, high salt content, months of darkness, excessive solar radiation and nutrient and carbon restrictions [6], [7], [8], [9]. Only those species with specific physiological adaptations have been able to survive under such extreme conditions, and this has been hypothesised as one of the main reasons for a depauperate soil microfaunal community [9], [10], [11]. Soil microfauna play an essential role in recycling nutrients and aiding decomposition, forming a vital component in Antarctic food webs [1], [12]. Low diversity food webs found in these soils ensure that nutrient recycling and trophic level interactions are restricted to microbial and metazoan invertebrate communities [13], [14]. It has been increasingly recognised that biotic soil communities are influenced by soil geochemical and physical properties [15], [16], [17], in particular organic carbon [1], [7], [18], conductivity [7], [9], and availability of liquid water [17], [19] as the main suggested drivers.

Even within ice-free areas, the distribution of microfaunal populations remains irregular and taxonomically limited [20], [21]. It remains unclear if these populations are limited by edaphic factors, microclimatic conditions, vegetation, or topography (e.g. [22]), with more abundant and diverse communities usually occurring in connection with patches of moss, lichens, algae [9], [11] and bird colonies [23], [24]. Rotifers, nematodes, tardigrades, protozoans [25], [26], [27] and, to a lesser extent, mites and springtails [28] make up the invertebrate communities of soil microfauna in East Antarctica (EA). Invertebrates are patchily distributed in soil and vegetation from ice-free areas in coastal and continental Antarctica and inland nunataks (exposed ridges or mountain peaks) [24], [29], [30], [31], [32]. Recent studies revealed that several Antarctic localities remained ice-free throughout the Last Glacial Maximum [9], [33] and that many terrestrial habitats are likely to have only become available for colonisation from refuges within the current inter-glacial period (<17,000 years) [9], [34]. However, there is compelling evidence that some regions are likely to have been ice-free for much longer and so it is likely that there exists an Antarctic terrestrial invertebrate fauna that consists of descendants from Gondwanan times. These have diversified in isolated ice-free locations since the completion of glaciation within the late Miocene (at approximately 21 to 11 Myr; e.g. [35], [36]).

Studies of ice-free areas across Antarctica have shown variations in microfaunal composition according to location and habitat. Microfaunal abundance also shows seasonal variation with respect to abiotic factors. Higher moisture content in soil during summer, as a consequence of higher temperatures, has been associated to increase the growth of photosynthetic autotrophs, microbial and microfaunal species [37]. Vertical distribution of microfauna in the soil profile has also been recorded to be affected by seasonal changes, with temperature and food source as likely determining factors [15]. For example, nematodes have been identified as the most diverse and abundant invertebrate group from Victoria Land [38], [39]; contrasting with results from Dronning Maud Land that have revealed rotifers, followed by tardigrades and nematodes as the most common taxa [11], [40], [41]. When considering the diversity of microfauna in soil, competition should also be expected to influence community structure – some studies have identified nematodes as the top grazers [1], while others reported competition among nematodes, rotifers, tardigrades and ciliates, and in some cases tardigrades and mites preying on nematodes [42].

Given the diversity of nematodes, a number of studies have focused their attention on the identification of Antarctic species. A total of 22 nematode species for continental Antarctica have been recorded and at least 90% of them are endemic [43], [44]. Some of the most common species recorded for the continent are the microbial feeders *Plectus murrayi*, *P. frigophilus* [27], [45], [46], *Scottnema lindsayae* [47], the omnivore genus *Eudorylaimus* [27], [44], [48], and the bacterial feeder *Panagrolaimus* [10], [40]. Other nematodes, occurring in lower abundance in EA, include the genus *Halomonhystera* [43], [49], *Hypodontolaimus* [43], and *Dolichorhabditis* [50]. Studies of tardigrades in EA have recorded 18 species [51], [52], [53], [54], belonging to three Orders (Apochela, Parachela and Echiniscoidea). The Order Parachela includes 15 species in ten genera; and the remaining two Orders are represented by the tardigrade genera *Echiniscus* and *Pseudoechiniscus* (Echiniscoidea) and the predatory species *Milnesium tardigradum* (Apochela). For rotifers, the Classes Bdelloidea and Monogononta have been reported for the Antarctic continent [55], [56], with Bdelloidea being the most widespread and abundant invertebrate group for EA soils [40], [57], with 22 species belonging to the genera *Adineta*, *Habrotrocha*, *Macrotrachela*, *Mniobia, Otostephanos* and *Philodina* (e.g. [41], [56], [58], [59]). Unfortunately, most studies have limited spatial coverage (often opportunistic) and low sampling sizes to gauge if the true biodiversity is accurately represented in current records (Table 1). In particular, some areas in EA have revealed a lower than expected diversity; with the recorded species down to nine nematode, seven bdelloid rotifer, and 15 tardigrade species. More comprehensive studies covering not only the diversity but also taking into account the environmental micro-habitats will provide data that can allow more robust comparisons at broad geographic scales.

**Table 1:** Diversity list for nematodes, bdelloid rotifers and tardigrades from East Antarctica showing previous record from the sampled regions.

	MS-FM	VH-LH	CS
**NEMATODA**			
**Order Rhabditida**			
*Dolichorhabditis tereticorpus* Kito & Ohyama, 2008	-	-	50
*Scottnema lindsayae* Timm, 1971	nr	nr	-
**Order Plectida**			
*Plectus frigophilus* Kirjanova, 1958	nr	43	nr
*Plectus murrayi* Yeates, 1970	nr	60	72
**Order Dorylaimida**			
*Eudorylaimus glacialis* Andrássy, 1998	44	-	-
*Eudorylaimus quintus* Andrássy, 2008	-	44	-
*Eudorylaimus sextus* Andrássy, 2008	-	44	-
**Order Monhysterida**			
*Halomonhystera continentalis* Andrássy, 2006	-	49, 43	-
*Halomonhystera halophila* Andrássy, 2006	-	49, 43	-
**Order Desmodorida**			
*Hypodontolaimus antarcticus* Andrássy & Gibson, 2007	-	43	-
**Order Panagrolaimida** (family cf. Panagrolaimidae)	nr?	nr?	
**ROTIFERA**			
**Order Bdelloidea**			
*Adineta barbata* Janson, 1983	-	56	*
*Adineta grandis* Murray, 1910	*	56, 55	58
*Habrotrocha constricta* Dujardin, 1841	*	56, 55	58
*Macrotrachela quadricornifera* Milne, 1886	-	56	-
*Mniobia russeola* (Zelinka, 1891)	-	56	-
*Philodina gregaria* Murrayi, 1910	*	56, 55	58
*Philodina alata*	*	*	-
**TARDIGRADA**			
**Order Parachela**			
*Acutuncus antarcticus* (Binda & Pilato, 2000)	53	60, 83	54
*Diphascon chilenense* (Sudzuki, 1964)	-	83	54
*Diphascon (Diphascon) pingue* Marcus, 1936	-	-	54
*Diphascon (Diphascon?) puniceum* Jennings, 1971	*	29	-
*Diphascon sanae* Dastych, Ryan & Watkins, 1990	53	-	-
*Hypsibius allisoni* Horning, Schuster & Grigarick, 1978	-	29	-
*Macrobiotus blocki* Dastych, 1984	53	-	-
*Macrobiotus furciger* Murray, 1907	*	29	-
*Macrobiotus weinerorum* Dastych, 1984	-	83	-
*Minibiotus stuckenbergi* Dastych, Ryan & Watkins, 1990	53	-	-
*Ramajendas frigidus* Pilato & Binda, 1990	-	-	54
**Order Apochela**			
*Milnesium* cf. *tardigradum* Doyere, 1840	53	83	-
**Order Echiniscoidea**			
*Echiniscus jenningsi* Dastych, 1984	53	-	-
*Pseudechiniscus* cf. *suillus*	-	83	54
*Pseudoechiniscus novaezeelandiae* Richters, 1903	-	60, 29, 83	-

* Previously reported by John Gibson (unpublished data).

The list includes taxa (nematodes, bdelloid rotifers and tardigrades) reported in the literature for the regions: Mawson Station – Framnes Mtns (MS-FM), Vestfold Hills – Larsemann Hills (VH-LH), and Casey Station (CS, including the Windmill Islands). New records for the regions obtained in this study are indicated by ‘nr’ (new records are based on absence of published literature for the designated region). Symbol ‘?’ means uncertainty for the record. Numbers indicate reference source (as in reference list).

In this study, we investigate environmental variables, soil geochemistry, and abundance and diversity of soil microfauna from different habitat types in East Antarctic regions; from Holme Bay (67.60°S – 62.87°E) and Framnes Mountains (67.78°S– 62.79°E) to Bailey Peninsula (66.28°S-110.54°E). To the best of our knowledge this is the first single study that correlates biotic and abiotic parameters for an area spanning more than 2,000 km from any region in Antarctica. Other studies have focused on diversity at a much smaller scale (e.g. [11], [31], [60]) including those that have considered abiotic variables for other Antarctic regions [9], [13], [17], [61]. We examine four questions: (1) Do abiotic variables differ significantly among the sample sites?; (2) If abiotic variables differ among sites, which variables best correlate taxa composition among sites (and to what extent); (3) Is microfaunal abundance affected by soil geochemistry and other abiotic variables; and (4) Is the occurrence of taxa correlated with the presence of other taxa?

## Materials and Methods

### Sampling sites

**Figure 1:**
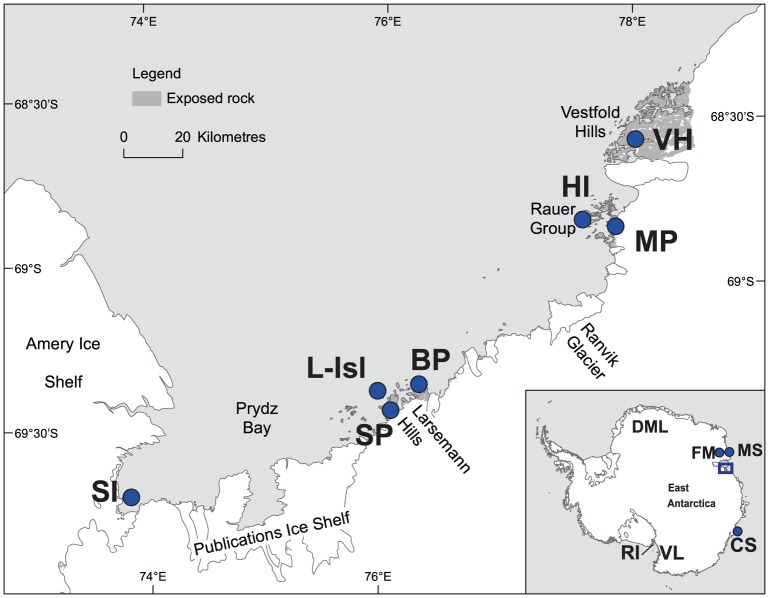
Maps showing the ten regions from East Antarctica (EA) where sampling was conducted (filled circles). Abbreviations: Framnes Mountains (FM), Mawson Station (MS), Casey Station (CS), Sansom Island (SI), Larsemann-Islands (L-Isl), Stornes Peninsula (SP), Broknes Peninsula (BP), Hop Island (HI), Mather Peninsula (MP), and Vestfold Hills (VH). Other sectors and regions across Antarctica mentioned in the text (not included in this study): Dronning Maud Land (DML), Victoria Land (VL), and Ross Island (RI). Adapted from maps provided courtesy of the Australian Antarctic Division.

All field activities and sampling in Antarctica was undertaken with permits granted by the Australian Antarctic Division (Australian Federal Government, Department of Sustainability, Environment, Water, Population and Communities). Samples returned to Australia under required quarantine protocols with permits granted by Australian Quarantine Inspection Service (AQIS, Australian Federal Government, Department of Agriculture, Fisheries and Forestry). Under these permitted guidelines, sampling in EA was conducted during the 2009–2010 austral summer from Casey Station on 24 December 2009, and from all other locations from 14 January 2010 to 4 March 2010 (Table S1 in File S1). Sampling locations were distributed over ten arbitrarily defined regions ranging from 67°–69°S to 62°–110°E with elevations ranging from 0 m to 490 m (Fig. 1). A total of 109 samples from ice-free areas were collected from ten regions: Casey Station (CS), Vestfold Hills (VH), Larsemann Hills-Broknes Peninsula (BP), Larsemann Hills-Stornes Peninsula (SP), Larsemann islands (L-Isl), Hop Island (HI), Mather Peninsula (MP), Sansom Island (SI), Framnes Mountains (FM) and Mawson Station (MS; Table 2). Sites were selected to represent a diversity of habitats with the intent of capturing a wide diversity of microinvertebrates; habitat types included visible vegetation (moss, cyanobacteria or algae), bird colonies and/or water bodies, and dry soils to semi-dry soil with no apparent vegetation. Soil core samples were excavated using a metal trowel carefully cleaned to avoid cross contamination. They were ∼10 cm in surface area, ∼10 cm deep (depth varied depending on the terrain) and 500–800 g wet weight. The top 10 cm were sampled as earlier studies have shown that throughout the summer season the majority of Antarctic soil microfauna inhabit this layer [15]. Samples were thoroughly mixed and kept in sterile 42 fl. oz. Whirl-pak® bags inside insulated containers while in the field and maintained at −20°C during storage and transit.

**Table 2:** Geographic location and type of samples collected from ten regions across East Antarctica.

REGION	Coordinates		Area sampled (km)	Elev (m)	Sample content			
	South	East			Soil-Gravel	Soil-al-cy	Soil-Moss	Total Samples
**Casey Station CS)**	66.28°	110.52°–110.54°	1×1.5	4–44	4	1	9	14
**Vestfold Hills (VH)**	68.48°–68.60°	77.87°–78.51°	17×20	4–66	11	5	6	22
**Broknes Peninsula (BP)**	69.38°–69.4°	76.32°–76.40°	3.5×2	0–69	13	1	0	14
**Stornes Peninsula (SP)**	69.37°–69.43°	75.99°–76.14°	6×1	4–59	4	1	4	9
**Larsemann Islands (L-Isl)**	69.36°–69.41°	76°–76.14°	7×0.1*	21–27	6	0	5	11
**Hop Island (HI)**	68.82°–68.83°	77.68°–77.73°	2×2	10–36	10	5	1	16
**Mather Peninsula (MP)**	68.85°–68.86°	77.93°–77.94°	1×1	44–80	1	1	4	6
**Sansom Island (SI)**	69.71°	73.75°	0.2×0.2	15–20	0	0	3	3
**Mawson Station (MS)**	67.60°	62.86°–62.87°	0.6×0.8	4–24	2	2	2	6
**Framnes Mountains (FM)**	67.77°–67.78°	62.79°–62.82°	3×1	460–490	6	0	0	6
**TOTAL**				0–490	57	18	34	107‡

* Two small islands 7 km apart. For the first Island (400 m south of Cook Island) samples were taken 25 m apart.

For the second (McLeod Island) samples were within 100 m^2^. Acronyms as following: Elevation (Elev), algae-cyanobacteria (al-cy).

‡ One sample from MP and other from MS included soil-lichen (not shown in the table).

### Microfaunal extraction, isolation and classification

Microfauna were extracted from the soil samples using a modified sugar centrifugation method [62]. Extractions were performed on 100 g soil samples (wet weight) after which stones larger than 1 cm were removed. Soil was poured onto a coarse sieve (400 µm mesh size) and carefully rinsed with double distilled water. The suspension of fine soil and water that flowed through the 400 µm mesh was kept in a tray 7 cm deep. This suspension was poured onto a 38 µm mesh and gently rinsed through (keeping the sieve at an angle of 30°). Water and fine sediment flowing through the 38 µm mesh was discarded. The fine soil retained on the 38 µm mesh sieve was washed into one or two 50 ml centrifuge tubes (depending on the quantity of fine soil, never exceeding 15 ml of soil per tube) and then topped up with water to 50 ml and mixed gently by inversion. Tubes were centrifuged at 500 RCF for 5 min, and the supernatant was decanted through a 38 µm mesh sieve (some animals retained in the sieve were recovered at this stage) whilst attempting to minimise the pouring out of any sediment onto the sieve. The tube was filled up with 1.3 M sucrose solution up to 50 ml and gently mixed by inversion to resuspend the pellet, and then centrifuged at 500 RCF for 1 min. The aqueous layer was then decanted into the 38 µm mesh sieve, again avoiding the transfer of any sediment from the pellet, and then back-washed into a clean 50 ml tube and storage at −20°C until further analysis.

Tubes containing microfauna in frozen distilled water were thawed and poured into a petri-dish to be examined under a dissecting stereo microscope (Olympus SZ-PT, Japan) at magnification 10× to 40×. Before isolation of specimens, presence of rotifers, nematodes, tardigrades, mites and ciliates were recorded and sorted coarsely within a gridded petri-dish. Individual specimens were then counted and abundance for each of the taxa assessed. In cases where samples were difficult to sort due to excessive amount of suspended material, further dilution was required. Samples with a high abundance of microfauna were sub-sampled and the total abundance was extrapolated for 100 g of soil.

Taxa were divided into glass blocks using modified gel tips attached to micro-syringes. Representative morphotypes for each taxon were retained for subsequent morphological analyses. Specimens were carefully transferred with an Irwin loop into a water droplet on a slide and imaged under microscope (Celestron- LCD Digital Microscope, USA) at 40× to 100× before placing in separate 2 ml Eppendorf tubes. The remaining microfauna not selected for imaging were stored at −20°C.

For the abundance analyses, all Rotifera were pooled into a single category, as was the case for Tardigrada, Nematoda, Ciliophora and Acari. For the taxa composition analyses (based on presence/absence), Ciliophora (ciliates) and Acari (mites) had their own separate categories, Rotifera were subdivided into monogononts (non-bdelloid rotifers) and bdelloids. Bdelloid identification was based on presence or absence of wheel-organs. The three bdelloid groups included *Philodina* (wheel-organ bearing bdelloid), *Adineta* (lacking wheel-organs) and a group including unidentified bdelloids (mostly comprising contracted specimens). Tardigrada were grouped according to their Order (Parachela, Apochela, Echiniscoidea); and Nematoda were categorised as *Plectus, Eudorylaimus, Scottnema, Halomonhystera* (genera) and cf. Panagrolaimidae (family). *Plectus* species were identified using de Man's ratios calculated from digital images (after [63]) and verified by comparison with published species descriptions (see Table S2 in File S1).

### Soil analyses

Soil geochemical analyses were performed for each of the 109 samples collected across EA. These analyses were conducted in Australia by APAL (Australian Perry Agricultural Laboratory) using standard chemical methods [64]. Subsamples of 100 g of soil were analysed for electrical conductivity (EC), organic carbon (C), Olsen-phosphorus (P), NO_3_^−^ and NH_4_^+^. Analyses for soil moisture (moist) and pH were performed at the University of Adelaide using the methods described by Rayment & Lyons [64]. Soil moisture was calculated from an average of 40 g of wet soil, and pH (pH meter- Schott® Instruments) was measured in a soil/water (1∶5) mixture at the University of Adelaide. The suspension was stirred constantly during the measurement to minimize changes in electrode potential. Other categories considered in our analyses included: fine sediment (amount of fine sediments in sample ranging between 38–400 µm), and particle size (qualitative gradient from silt to coarse gravel).

### Statistical Analyses

#### Defining abiotic categories

Environmental variables were elevation, aspect (direction to which the slope faces), slope, vegetation content in soil (visible moss, cyanobacteria, algae or lichen in sample), and proximity to moss beds when present. Other categories included region, geology of the terrain, amount of fine sediment in the sample and the soil geochemical parameters analysed (EC, C, P, NO_3_^−^, NH_4_^+^, moist and pH). In total, 16 (categorical) abiotic variables were considered, with ten of these quantitative and six qualitative. The categories moss, algae-cyanobacteria (al-cy), and soil samples from moss beds were qualitative dichotomous (i.e., presence/absence); the categories region, geology and aspect were qualitative. Regions included: CS, VH, BP, SP, L-Isl, HI, MP, SI, FM and MS. Geology of terrain comprised three sub-categories as reported by tectonic studies [65], [66]. It consisted on mainly archaean complexes (VH and CS); mixed archaean-proterozoic complexes (HI and MP); and mainly meso-neoproterozoic (BP, SP, SI, MS and FM). Aspect included three sub-categories (1, 2 and 0) representing north-facing, south-facing and east/west/flat-facing (respectively). North-west and north-east sites were merged under the sub-category north facing, and south-west and south-east merged under south facing.

#### Biotic and abiotic

Biotic and abiotic categories and the interaction between them were analysed using PRIMER v.6 [67]. Abiotic data for quantitative abiotic categories were logarithmically (base-10) transformed [68] to avoid right skewness (as detected using Draftman Plots before transformation) and a small constant was added (0.1) to avoid zero values (after [67]). Qualitative and log[0.1+*x*] transformed quantitative variables were normalised (for each entry of a single variable the mean is subtracted and divided by the standard deviation of that variable) and then subjected to a Principal Component Analysis (PCA) based on Euclidean distances (after [69], [70]) in order to identify the most relevant categories and the cumulative percentage variation of PCAs. Points on the PCA ordination plot were colour coded by region to place the analysis in a geographical context. A preliminary colinearity test for normalised abiotic categories based on the resemblance matrix of the Draftsman Plot was first estimated in order to reduce the amount of variables (after [67]). Only one category (geology) was dropped from the analysis given its strong colinearity with one other variable (region) as observed in the resemblance matrix (0.9 correlation value). The second strongest correlation (0.7) was seen for P and NH_4_^+^ but was not high enough to be excluded from the analyses (after [67]). PCA was used to indirectly correlate parameters (vectors) and sampling sites. Resemblance matrices (for biotic data) were created for taxa composition and 4^th^ root transformed microfauna abundance based on Bray-Curtis similarity coefficients to correct for skewness in the data to achieve normality [17], [68], [71]. Matrices on taxa composition were also used to generate individual hierarchical clusters for rotifers, tardigrades, nematodes, and (combined) microfaunal taxa (rotifers, tardigrades, nematodes, ciliates and mites). To correlate the relative contribution of abiotic variables with microfauna abundance and taxa composition the Bioenv method (PRIMER v.6) was employed using the Spearman correlation coefficient (after [67]).The Pearson correlation method was also used to correlate biotic and abiotic variables using IBM-SPSS Statistics v19 (see Table S3 in File S1).

## Results

### Environmental assemblages

**Figure 2:**
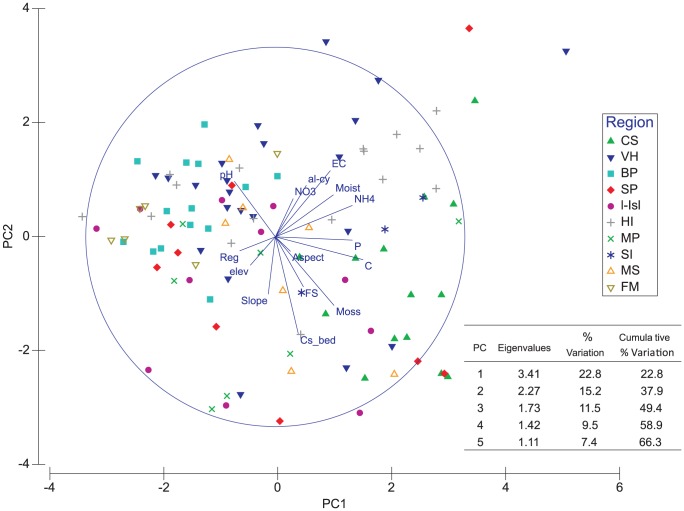
Principal component analysis (PCA) on log [x+0.1] transformed and normalized values of abiotic data from 109 sites. Symbol shapes represent region for each of the samples. Vectors labelled as region (Reg), elevation (elev), soil sample from moss bed (Cs_bed), fine sediment (FS), samples with moss filaments (moss), aspect, organic carbon (C), Olsen-phosphorus (P), NH_4_^+^, moisture in soil (Moist), electrical conductivity (EC), samples containing alga-cyanobacteria (al-cy), NO_3_^−^, and pH.

**Figure 3:**
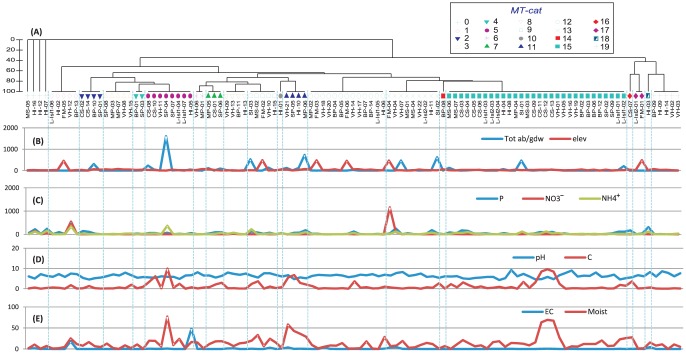
*(A)* Hierarchical cluster of taxa composition based on Bray-Curtis similarity coefficient (presence/absence of microfaunal taxa). *(B)* Microfauna total abundance given in grams of dry weight of soil (Tot ab/gdw); and elevation at which samples were collected. *(C–E)* values for soil geochemical variables for 109 samples across EA. Geochemical variables (units and acronyms): Olsen-phosphorus ‘P’ (mg/kg), NH_4_^+^ (ppm), NO_3_^−^ (ppm), soil moisture ‘Moist’ (%), electric conductivity ‘EC’ (ds/m), and organic carbon ‘C’ (%). The Order of samples for graphs *(B–E)* is the same as indicated in cluster *(A)*. Color-coded symbols identified by the Hierarchical cluster (separated by blue dotted line) represent microfaunal taxa categories (*MT-cat*): ‘0’ no microfauna, ‘1’ rot, ‘2’ rot-mit, ‘3’ rot-nem-mit, ‘4’ rot-tar-mit, ‘5’ rot-nem-tar-mit, ‘6’ rot-nem-cil-mit, , ‘7’ rot-nem-tar-cil-mit. ‘8’ rot-nem-cil, ‘9’ rot-nem-tar-cil, ‘10’ rot-cil, ‘11’ rot-tar-cil, ‘12’ rot-nem ‘13’ rot-tar, ‘14’ tar-nem ‘15’ rot-nem-tar, ‘16’ mit, ‘17’ nem-mit, ‘18’ nem-cil, and ‘19’ nem. Abbreviations used: rotifers (rot), tardigrades (tar), nematodes (nem), mites (mit), and ciliates (cil).

**Figure 4:**
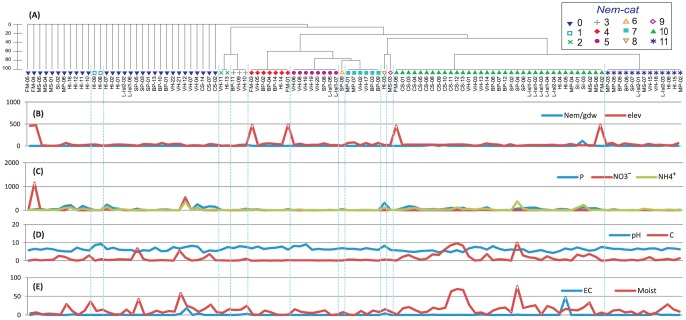
*(A)* Hierarchical cluster of nematode composition based on Bray-Curtis similarity coefficient (presence/absence of morphologically identified taxa). *(B)* Nematode total abundance given in grams of dry weight of soil (Nem/gdw); and elevation at which samples were collected. *(C–E)* values for soil geochemical variables for 109 samples across EA. Geochemical variables (units and acronyms): Olsen-phosphorus ‘P’ (mg/kg), NH_4_^+^ (ppm), NO_3_^−^ (ppm), soil moisture ‘Moist’ (%), electric conductivity ‘EC’ (ds/m), and organic carbon ‘C’ (%). The Order of samples for graphs *(B–E)* is the same as indicated in cluster *(A)*. Color-coded symbols identified by the Hierarchical cluster (separated by blue dotted line) represent nematode categories (*Nem-cat*): ‘0’ no nematodes, ‘1’ undetermined, ‘2’ Ha-Sc, ‘3’ Ha, ‘4’ Sc, ‘5’ Sc-Eu, ‘6’ Sc-Pt, ‘7’ Pt-Eu-Sc, ‘8’ Pa, ‘9’ Pt-Pa, ‘10’ Pt, and ‘11’ Eu-Pt. Abbreviations used: *Plectus* (Pt), *Halomonhystera* (Ha), cf. Panagrolaimidae (Pa), *Scottnema* (Sc), and *Eudorylaimus* (Eu).

**Figure 5:**
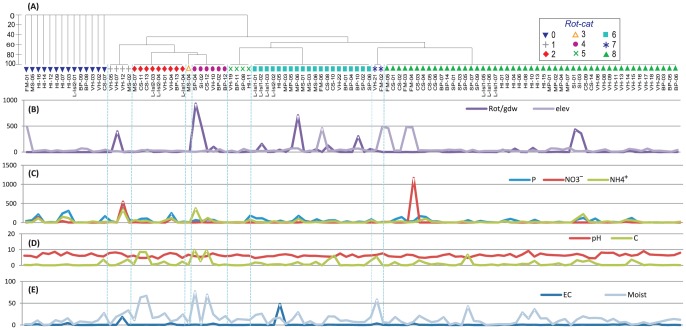
*(A)* Hierarchical cluster of rotifer composition based on Bray-Curtis similarity coefficient (presence/absence of morphologically identified taxa). *(B)* Rotifer total abundance given in grams of dry weight of soil (Rot/gdw); and elevation at which samples were collected. *(C–E)* values for soil geochemical variables for 109 samples across EA. Geochemical variables (units and acronyms): Olsen-phosphorus ‘P’ (mg/kg), NH_4_^+^ (ppm), NO_3_^−^ (ppm), soil moisture ‘Moist’ (%), electric conductivity ‘EC’ (ds/m), and organic carbon ‘C’ (%). The Order of samples for graphs *(B–E)* is the same as indicated in cluster *(A)*. Color-coded symbols identified by the Hierarchical cluster (separated by blue dotted line) represent rotifer categories (*Rot-cat*): ‘0’ no rotifers, ‘1’ Ph, ‘2’ Ph-ub, ‘3’ Ph-Ad, ‘4’ Ph-Ad-ub, ‘5’ Ad, ‘6’ Ad-ub, ‘7’ ub-Monogonota, and ‘8’ ub. Abbreviations used: *Adineta* (Ad), *Philodina* (Ph), and unidentified bdelloid (ub).

**Figure 6:**
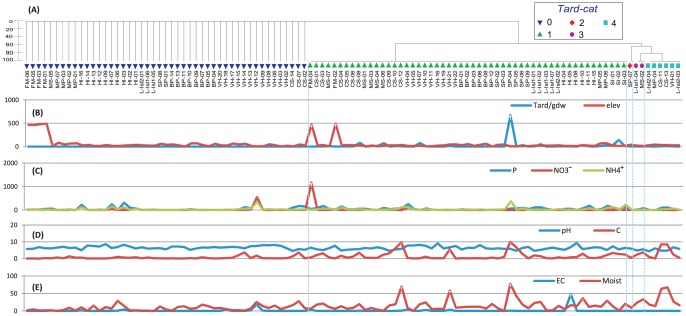
*(A)* Hierarchical cluster of tardigrade composition based on Bray-Curtis similarity coefficient (presence/absence of morphologically identified taxa). *(B)* Tardigrade total abundance given in grams of dry weight of soil (Tar/gdw); and elevation at which samples were collected. *(C–E)* values for soil geochemical variables for 109 samples across EA. Geochemical variables (units and acronyms): Olsen-phosphorus ‘P’ (mg/kg), NH_4_^+^ (ppm), NO_3_^−^ (ppm), soil moisture ‘Moist’ (%), electric conductivity ‘EC’ (ds/m), and organic carbon ‘C’ (%). The Order of samples for graphs *(B–E)* is the same as indicated in cluster *(A)*. Color-coded symbols identified by the Hierarchical cluster (separated by blue dotted line) represent tardigrade categories (*Tard-cat*): ‘0’ no tardigrades, ‘1’ Parachela, ‘2’ Echiniscoidea, ‘3’ Parachela-Apochela-Echiniscoidea, and ‘4’ Parachela-Echiniscoidea.

The PCA is presented in Figure 2, with vector length indicating the relevance of the abiotic variable in question, and the orientation of the vector showing the positive or negative influence in reference to the cluster of sites. The most significant abiotic variables (indicated by vector length) corresponded to C, soil samples from moss beds (Cs_bed), samples containing moss filaments (moss), NH_4_^+^, EC, P, pH and moist; and the least significant variable was aspect. The distribution of samples among regions was better explained by PC1 (as observed for BP, FM and CS). CS samples were segregated to the right of PC1 (showing positive correlations to C, P and moss); BP and FM tend to segregate along the PC1 axis to the left of the cluster (negative values). No clear trend was observed for the other regions along PC1 and PC2 axes (Fig. 2). Overall, PC1 explained 22.8% of the variation among environmental variables. The cumulative variation of PC1–PC2 was 37.9% and PC3–PC5 had a cumulative variation of 28.4%. The vectors for logarithmic transformed variables (C, P and NH_4_^+^) were positively correlated and presented the highest contribution for PC1 (eigenvectors: 0.46, 0.41 and 0.41, respectively). For PC2 the highest contribution was observed for Cs_bed, moss and by EC (eigenvectors: −0.51, 0.36 and 0.35, respectively). Cs_bed was positively correlated to moss samples but negatively correlated with pH. When examining other type of vegetation, we observed that algae-cyanobacteria (al-cy) was better explained by PC2 (eigenvectors: 0.27). A positive correlation was also observed between al-cy and NO_3_^−^, but a negative correlation with elevation (Fig. 2). To identify groups of correlated abiotic variables some of the associations between soil abiotic parameters indicated by the PCA are corroborated with results summarized in the Pearson correlation matrix (Table S3 in File S1) and geochemical parameters (Figs. 3–6). In general, when considering soil geochemical parameters positive correlations were seen for: *(i)* EC and NO_3_^−^; *(ii)* P, NH_4_^+^, C and soil moisture.

### Taxa composition and terrestrial habitats

Taxa composition data (absence/presence) for 109 samples from the ten regions (Table 3) were used to identify closely related clusters based on Bray-Curtis similarity coefficient. Four hierarchical clusters were generated, one including all microfaunal taxa (rotifers, tardigrades, nematodes, ciliates and mites) as observed for each soil sample (Fig. 3A). The three other clusters represented taxa composition categories found for nematodes (Fig. 4A), rotifers (Fig. 5A), and tardigrades (Fig. 6A). Bdelloid rotifers were the most widespread taxon present in 87% of the samples, followed by nematodes in 71%, tardigrades in 57%, mites (including mite exuviae) in 23%, and ciliates in 15% (Table 3e). The presence of all microfauna taxa (combined) only occurred in three soil samples from CS, MP and SP (Fig. 3A). These three samples were from moss beds, with visible moss filaments and were characterised by high moisture (12–18%), wide ranges of C (0.8–2.9%), P (38–92 p.p.m.), and NH_4_^+^ (5–64 p.p.m.), and low NO_3_^−^ (3.4 p.p.m.) and EC (0.06–0.15 dS/m; Fig. 3). The presence of the three most common taxa (rotifers, tardigrades and nematodes) in the absence of ciliates and mites were found for 30 samples (Fig. 3A). Microfauna was absent in four samples (all with no visible vegetation); three of which were from Hop Island (Fig. 3), and two collected next to bird colonies. Rotifers (bdelloids) occurred as a single taxon in five samples, with a wide range of soil geochemical properties: EC (0.02–18.5 dS/m), NO_3_^−^ (3.4–548 p.p.m.), NH_4_^+^ (4.8–345 p.p.m.), and P (4.5–469 mg/kg; Fig. 3). Nematodes were also found as the only taxon in five samples (without visible vegetation), but under more restricted concentrations of NO_3_^−^ (3.4 p.p.m), C (0.01–0.22), EC (0.02–0.1 dS/m), and P (3.6–11.6 mg/kg; Fig. 3).

**Table 3:** Sample size (a), Taxa absent (b), Abundance (c), Percentage of Abundance (d), and taxa composition percentage (e) of microfauna from 109 soil samples at ten regions.

	All sites	CS	VH	HI	MP	LH-BP	LH-SP	L-Isl	SI	MS	FM
**(a) Sample size (number of samples collected)**											
	109	14	22	16	7	14	9	11	3	7	6
**(b) Taxa absent (number of samples with no visible taxa)**											
	4	0	0	3	0	0	0	0	0	1	0
**(c) Abundance (average number of animals/g dry weight on occurrence)**											
Rotifera	4756.3	264.3	576.8	244	802.8	312	1010.2	191.3	1279.3	70.5	5.1
Tardigrada	1363	113.6	101.3	42.7	35.1	8.2	711.8	57.1	183.5	109.5	0.1
Nematoda	326.4	58.5	75	16.7	20.2	3.7	7.9	7.6	120.7	13.3	2.8
Ciliophora	139.8	0.8	88.5	7.5	1	0	0.4	0	41.4	0	0.1
Acari	3.9	0.5	0.8	0.3	0	0.1	2	0.1	0	0	0
***Total***	**6589**	**438**	**842**	**311**	**859**	**324**	**1732**	**256**	**1625**	**193**	**8**
***Average***	**60**	**31**	**38**	**19**	**123**	**23**	**192**	**23**	**542**	**28**	**1**
**(d) Percentage of Abundance**											
Rotifera	**72.18**	60.37	68.47	78.43	93.44	96.29	58.32	74.68	78.73	36.48	63.34
Tardigrada	**20.68**	25.96	12.02	13.72	4.09	2.54	41.09	22.3	11.3	56.65	0.92
Nematoda	**4.95**	13.36	8.91	5.35	2.35	1.14	0.46	2.97	7.43	6.86	34.56
Ciliophora	**2.12**	0.19	10.51	2.41	0.12	0.01	0.02	0	2.55	0.01	1.05
Acari	**0.06**	0.12	0.09	0.09	0	0.02	0.12	0.06	0	0	0.13
**(e) Taxa composition percentage (based on presence-absence)**											
**Rotifera**	87.2	11.9	18.3	9.2	6.4	11	8.3	9.2	2.8	5.5	4.6
Monogononta	0	0	0.92	0	0	0	0	0	0	0	0.92
Unident-Bdell	78.9	11.9	14.7	8.3	6.4	10.1	7.3	9.2	2.8	3.7	4.6
*Adineta*	28.4	2.8	2.8	1.8	2.8	5.5	3.7	3.7	0.9	3.7	0.9
*Philodina*	18.3	2.8	5.5	0.0	0.0	2.8	0.9	2.8	0.9	2.8	0.0
**Tardigrada**	56.9	10.1	9.2	5.5	2.8	5.5	6.4	7.3	2.8	5.5	1.8
Parachela	56	10.1	9.2	5.5	2.8	5.5	5.5	7.3	2.8	5.5	1.8
Apochela	1.8	0	0	0	0	0	0	0.9	0	0.9	0
Echiniscoidea	8.3	1.8	0.9	0	0.9	0	0.9	2.8	0	0.9	0
**Nematoda**	71.6	10.1	15.6	7.3	5.5	10.1	5.5	8.3	1.8	3.7	3.7
*Plectus*	51.4	10.1	7.3	3.7	5.5	6.4	4.6	6.4	1.8	3.7	1.8
*Eudorylaimus*	24.8	0	8.3	1.8	4.6	3.7	1.8	3.7	0	0.9	0
*Scottnema*	22	0	8.3	1.8	1.8	4.6	1.8	1.8	0	0	1.8
*Halomonhystera*	4.6	0	2.8	0.9	0	0.9	0	0	0	0	0
Panagrolaimidae	1.8	0	0	0.9	0	0	0	0	0	0.9	0
**Ciliophora**	15.6	0.9	4.6	4.6	1.8	0.9	1.8	0	0.9	0.9	0.9
**Acari** †	22.9	5.5	3.7	0.9	3.7	1.8	5.5	2.8	0	0	0.9

† Including 27 samples (20 with mite specimens, and 7 with only mite ecdysis).

Total abundance and average for the regions in **(c)** are given in bold. Percentage of abundance for all sites in **(d)** is shown in bold in 1^st^ column. Taxa composition in **(e)** refers to presence of taxa in samples (no abundance data considered for this category). List of acronyms: Casey Station (CS), Vestfold Hills (VH), Hop Island (HI), Mather Peninsula (MP), Larsemann Hills-Broknes Peninsula (BP), Larsemann Hills-Stornes Peninsula (SP), Larsemann - Islands (L-Isl), Sansom Island (SI), Mawson Station (MS), Framnes Mountains (FM), and Unidentified Bdelloids (Unident-Bdell).

#### Nematode composition and habitats

All nematodes were identified using morphological measurements and de Man's ratios. For *Plectus* morpho-types were compared with described populations for *Plectus murrayi* and *P. frigophilus* from continental Antarctica (Table S2 in File S1). Our study revealed the genus *Plectus* to be the most widespread, present in 51% of all 109 samples, followed by *Eudorylaimus* in 25%, *Scottnema* in 22%, *Halomonhystera* in 4.6% and cf. Panagrolaimidae in 1.8% of samples (Table 3e). *Plectus* occurred as the only nematode in 35 samples, followed by *Scottnema* (seven samples), *Halomonhystera* (three samples), and cf. Panagrolaimidae (one sample); while *Eudorylaimus* was always found in the presence of other nematode genera (Fig. 4A).The genus *Plectus*, was the only nematode genus present in all ten sampled regions (Fig.1, Table 3e). Although *Plectus* has been reported for EA [48], [50], [72] there are no published records for MS and FM (Table 1). *Plectus* was present in a wide range of environmental conditions (Fig. 4); with *P. murrayi* as the only nematode species observed from CS. *Plectus murrayi* was observed in samples with various ranges of C (0.01–9.9%), EC (0.01–48 dS/m), NH_4_^+^ (4.2–372 p.p.m.), NO_3_^−^ (3.4–19 p.p.m.), P (2–171 mg/kg), pH (4.3–8) and moisture (0.25–77%). *Plectus frigophilus* was less tolerant of extreme conditions as *P. murrayi*, occurring in only five sites with no visible moss filaments, limited EC range (0.04–0.88 dS/m), and diverse ranges of C (0.05–9.9%), NO_3_^−^ (3.4–12 p.p.m.), NH_4_^+^ (5.1–372 p.p.m.), pH (4.7–7.6), P (7.3–99 mg/kg) and moisture (6.5–77%). The minimum soil moisture requirements for *Plectus* species were higher than for *Scottnema* and *Eudorylaimus*.

*Scottnema* specimens were collected from VH to FM (Fig. 1) in seven of the sampled regions (Table 3e). Records for this genus comprised the first records in these regions (Table 1). *Scottnema* was present in 30% of the nematode samples and always in environments of low EC (0.02–0.38 dS/m), NH_4_^+^ (4.5–18.6 p.p.m.), and C (0–0.55%), at low-moderate levels of moisture (0.1–15.4%), and various levels of P (3–44 mg/kg) and NO_3_^−^ (18.4–3.4 p.p.m.). No visible cyanobacterial samples were associated with the presence of *Scottnema*. In samples where *Scottnema* was present, 58% of the time (14 samples) it occurred with *Eudorylaimus* (Fig. 4A) but never in the presence of the tardigrade *Echiniscus*. Soil geochemical variables seemed to be broader (in most cases) for *Eudorylaimus* than *Scottnema* (Fig. 4). *Eudorylaimus* was found from VH to MS (Fig.1, Table 3e) in soils of low-medium ranges of C (0.01–1.94%), and various levels of EC (0.01–3.5 dS/m), NH_4_^+^ (4.2–63.6 p.p.m.), NO_3_^−^ (3.4–11 p.p.m.), moisture (0.11–28.6%), and P (1.4–40 mg/kg).

*Halomonhystera* was found in a total of five samples in VH, BP, and HI (Table 3e), and occurred as the only nematode genus in three of them (Fig. 4A). It was never observed co-occurring with *Plectus* or *Eudorylaimus* (Fig. 4A), but with bdelloids and ciliates in 80% of samples. *Halomonhystera* occurred mostly in coarse gravel samples with no visible moss filaments, low C (0.05–1.08%), moderate NO_3_^−^ (3.4–7.5 p.p.m.), and various ranges of EC (0.04–3.02 dS/m), NH_4_^+^ (7–31 p.p.m.), moisture (5.8–24%), and P (4–67 mg/kg). Members of the family Panagrolaimidae have been recorded for EA [10], but as far as we are aware there are no previous records for the family in any of the ten sampled regions (Fig. 1). We found cf. Panagrolaimidae nematodes in two fine soil samples from HI and MS, and were the only nematode taxon from an ornithogenic soil (Fig. 4). Only one isolated specimen from a different genus (cf. *Hypodontolaimus*) was observed in a fine soil sample (HI-08) next to a saline lake (EC: 0.33 dS/m) without visible vegetation.

#### Rotifer composition and habitats

We identified the Classes Bdelloidea and Monogononta from our Antarctic soils. It was only possible to morphologically discern live-mobile bdelloid specimens (which constitute less than one third of all specimens). Seven bdelloid species have been previously described in the literature for VH, Larsemann Hills, and CS [55], [56], [58] (Table 1). We were able to discern the genera *Adineta* and *Philodina* from some of the samples, but the remaining bdelloids were left as unidentified.. Bdelloids were present for all ten regions (Table 3) in soil samples varying in particle size from fine to coarse, with and without vegetation; and in the most extreme conditions in a variety of geochemical ranges: EC (0.01–48 dS/m), C (0–9.9%), P (1.4–469 mg/kg), NO_3_^−^ (3.4–1163 p.p.m.), NH_4_^+^ (4.5–373 p.p.m.), moisture (0.11–77%) and pH (4.3–9.2; Fig. 5). The rotifer cluster (Fig. 5A) revealed nine categories, with the most common consisting of exclusively unidentified bdelloids (49 samples) in a single clade, followed by an ‘unidentified bdelloids-*Adineta*’ clade comprising 20 samples. *Philodina* and *Adineta* were found together in seven samples; while Monogononta (*Encentrum* cf., *Cephalodella* cf. and *Lepadella* cf.) was only observed in two samples from the sides of lakes with similar NH_4_^+^ concentrations (7.5–7.8 p.p.m.) and close to neutral pH (6.7–7.6).

#### Tardigrade composition and habitats

Three Orders of tardigrades (Parachela, Apochela and Echiniscoidea) were identified in this study (Table 1). In samples with tardigrades, Parachela was the most dominant and present in all samples (except one) distributed across a broad type of habitats (Fig. 6). Parachela was present within the same extended NO_3_^−^, NH_4_^+^ and pH ranges as bdelloids, but in a narrower range of: EC (0.02–48 dS/m), C (0.01–9.9%), P (1.9–249 mg/kg), and moisture (0.28–77). Parachela was recorded from 56% of the 109 samples followed by Echiniscoidea 8% and Apochela 1.8% (Table 3e). Apochela (represented by *Milnesium* sp.) occurred in two samples from L-Isl and MS together with the other two tardigrade Orders (Fig. 6), nematodes (*Plectus*) and rotifers. *Milnesium* was found in fine soils containing visible moss filaments, high moisture (24–33%) and P (27–79 mg/kg), moderate organic C (2.4–3.6%) and NH_4_^+^ (11–13 p.p.m.), and low pH (4.8–5.5). Echiniscoidea (represented by *Echiniscus* sp.) was present in nine samples (Fig. 6A) with different size soil particles, acidic pH (4.1–6.6) and no visible al-cy. All samples including *Echiniscus* also contained bdelloid rotifers and *Plectus*.

#### Ciliate composition and habitats

Ciliates were not further classified and left as un-identified morpho-types. The exception was the morpho-species *Paradileptus* cf. *elephantinus* which was observed in a single soil sample collected at a bird moulting site in HI. Ciliates were observed in seven regions (Table 3), and occurred in a range of habitats from fine to coarse soil size, in dry to wet conditions (moisture: 1.55–58.4%), in presence and absence of vegetation; in soils with low to moderate ranges of EC (0.04–4.4 dS/m), and NO_3_^−^ (3.4–41.5 p.p.m.); and soils with a wide range in C (0.05–6.8%), P (1.9–310 mg/kg), NH_4_^+^ (4.8–222 p.p.m.), and pH (4.7–8.24; Fig. 3).

#### Mite composition and habitats

The arthropod community in our EA soils was dominated by Prostigmata mites (cf. *Nanorchestes*, cf. *Tydeus*, and cf. *Stereotydeus*). They were found in seven of the ten geographic regions (Table 3) in a broad range of habitats (Fig. 3), from silty to coarse soils, in presence or absence of visible vegetation, and in soils presenting a wide range of EC (0.02–48 dS/m), pH (4.8–8.1), and C (0.01–6.14%) values; low to moderate values for P (3.9–169 mg/kg), NH_4_^+^ (4.5–64 p.p.m.), moisture (1.8–27.7%); and low NO_3_^−^ concentrations (3.4–8.2 p.p.m.), which was corroborated by a negative correlation for NO_3_^−^ and mite presence (Table S3 in File S1). One of the seven samples (LH-SP-04; Fig. 3) where mites were absent but mite exuviae present, was outside the maximum range observed for C, NH_4_^+^ and moisture values. Mites occurred in absence of any other taxa in one sample (CS-07; Fig. 3), which corresponded to the lowest NH_4_^+^ (18.9 p.p.m.), and the second highest P concentration (169.3 mg/kg) for all CS samples.

### Microfaunal abundance and vegetation

The average invertebrate abundance for the microfaunal taxa in EA soils were 60 specimens per gdw, with the highest average of specimens per region found in SI (542 specimens/gdw); and the lowest for FM (Table 3c). The most abundant taxon was rotifers, representing 72.2% of all invertebrates (average per sample: 44 specimens/gdw); followed by tardigrades 20.7% (12 specimens/gdw); nematodes 5% (3 specimens/gdw); ciliates 2% (1.3 specimens/gdw), and mites 0.06% (0.04 specimens/gdw; Table 3d). Abundance varied greatly among samples (Figs. 3B–6B). In 33% of the samples, it was less than 1 specimen/gdw; in 36% of samples, it ranged from 1–10 specimens/gdw, and in 13% of samples it was over 100 specimens/gdw (Table S1 in File S1).

From 109 samples, a total of 44 were identified with vegetation (moss, algae, cyanobacteria and/or lichen), which accounted for 82% of the total microfaunal abundance. There were 12 samples containing only al-cy as the only visible type of vegetation and accounted for 38% of microfauna abundance. Soil samples including moss (without visible al-cy) were 26 and represented 29% of the abundance (two of those sample also contained traces of lichens). Four samples included al-cy and moss (together) and contributed 14% of the abundance. Only two samples had lichen as the only form of vegetation and represented 2% of the abundance. Samples without visible vegetation (65 out of 109) included only 18% of the total microfauna abundance. Around 70% of the microfauna abundance was concentrated in six samples (Table S2 in File S1), with a single high moisture (77%) cyanobacteria sample from a lake edge (LH-SP-04; Fig. 3B–6B) accounting for 24% of the total invertebrate abundance (49% of tardigrades and 20% of rotifers). In the case of ciliates, 61% of their total recorded density occurred in a single sample rich in cyanobacteria flakes and 58% of moisture content (VH-21; Fig.3B). For nematodes (*Plectus*), a soil sample with visible moss filaments from Sansom Island (SI-03; Fig. 4B) contained 35% of the total nematode density. Similarly, for mites, 31% of their entire density occurred in a single sample with moss filaments and moderate moisture content (9.2%) from Stornes Peninsula (LH-SP-07; Fig. 3).

### Linkage between biotic and environmental parameters

Bioenv analyses were used to find the best combination for abiotic with biotic categories (taxa abundance and composition). For our study it was observed that the highest correlation among abiotic categories and taxa composition was a combination of P, NO_3_^−^, soil moisture and elevation (ρ = 0.221; Table 4). NH_4_^+^ (ρ = 0.126) followed by P (ρ = 0.108) and EC (ρ = 0.094) represented the abiotic variables with strongest correlations when considering each individually (Table 4). For microfauna total abundance it was observed that EC (ρ = 0.204), C (ρ = 0.198) and NH_4_^+^ (ρ = 0.186) presented the highest correlation values when considered individually; while a combination of C and NO_3_^−^ showed the highest two-variables correlation (ρ = 0.326; Table 4). The Bioenv analyses revealed that the best variables to explain nematode composition (all taxa combined) were al-cy (ρ = 0.15) and NO_3_^−^ (ρ = 0.149); while abundance was better explained by NO_3_^−^ (ρ = 0.206). A combination of P, NO_3_^−^, pH and al-cy had the strongest correlation values with nematode composition (ρ = 0.309); while the combined effect of P, NO_3_^−^ and al-cy had the highest correlation with nematode abundance (ρ = 0.335; Table 5). Considering the most frequent nematode genera separately (*Plectus*, *Eudorylaimus* and *Scottnema*) we observed that the strongest correlations for *Plectus* were NO_3_^−^ (ρ = 0.128) and al-cy (ρ = 0.149); for *Eudorylaimus*, P (ρ = 0.272), pH (ρ = 0.206) and C (ρ = 0.179); and for *Scottnema*, C (ρ = 0.283), NO_3_^−^ (ρ = 0.213) and NH_4_^+^ (ρ = 0.19; Table 5).

**Table 4:** Result from Bioenv analysis showing the strongest correlations for abiotic variables (when considered individually or in connection to others) that best match the biotic matrices for microfauna total abundance and composition.

Taxa composition			Meiofauna total abundance		
Number of Variables	Correlation (ρ)	Selection of Variables	Number of Variables	Correlation (ρ)	Selection of Variables
1	**0.126**	4	1	**0.204**	1
1	0.108	3	1	0.198	2
1	0.094	1	1	0.186	5
4	**0.221**	3,4,6,9	1	0.16	3
3	**0.219**	3,4,6	1	0.129	4
2	**0.205**	4,6	4	**0.328**	1,2,4,5
3	0.204	4,6,9	4	0.327	2,4,5,13
4	0.2	1,3,4,6	2	**0.326**	2,4
3	0.195	3,4,9	3	**0.323**	2,4,5
4	0.195	1,4,6,9	4	0.317	2,4,5,7
4	0.194	3,4,6,7	4	0.316	2,3,4,5

Numbers in bold indicate best correlation values for the selected combinations of abiotic variables. Numbers under Selection of Variables correspond to: electrical conductivity ‘EC’ (1), organic carbon ‘C’ (2), Olsen-phosphorus ‘P’ (3), NO_3_^−^ (4), NH_4_^+^ (5), Moist (6), pH (7), elevation (9), and algae-cyanobacteria (13).

**Table 5:** Result from Bioenv analysis showing the strongest correlations for abiotic variables (when considered individually or in connection to others) that best match the biotic matrices for nematode composition (all taxa combined, *Plectus*, *Eudorylaimus* and *Scottnema*), and abundance.

Nematode composition												Nematode abundance		
Nematod taxa (all)			*Plectus*			*Eudorylaimus*			*Scottnema*					
No.V	Corr. (ρ)	Sel.V	No.V	Corr. (ρ)	Sel.V	No.V	Corr. (ρ)	Sel.V	No.V	Corr. (ρ)	Sel.V	No.V	Corr. (ρ)	Sel.V
1	0.15	13	1	0.128	4	1	0.272	3	1	0.283	2	1	0.206	4
1	0.149	4	1	0.107	13	1	0.206	7	1	0.213	3	1	0.182	1
1	0.142	3	1	0.083	7	1	0.179	2	1	0.19	5	1	0.178	13
1	0.119	7	4	0.233	4,7,9,13	1	0.144	5	1	0.164	13	1	0.116	3
1	0.107	1	4	0.229	4,6,7,13	4	0.36	2,3,7,9	4	0.353	2,3,5,13	3	0.335	3,4,13
4	0.309	3,4,7,13	4	0.222	4,7,10,13	4	0.357	2,3,4,7	4	0.351	2,5,7,13	4	0.325	3,4,11,13
4	0.299	4,7,9,13	3	0.22	4,7,13	4	0.355	3,4,7,9	3	0.351	2,5,13	4	0.321	1,3,4,13
4	0.294	3,4,9,13	4	0.214	3,4,7,13	4	0.347	2,3,4,9	4	0.349	1,2,5,13	4	0.318	2,3,4,13
4	0.292	2,4,7,13	4	0.21	4,6,7,9	3	0.34	3,4,7	3	0.348	2,3,13	4	0.311	3,4,5,13
3	0.289	4,7,13	4	0.21	4,6,9,13	3	0.339	3,7,9	4	0.345	2,3,7,13	4	0.311	3,4,12,13
3	0.277	3,4,13	4	0.21	2,4,7,13	4	0.333	3,4,7,10	4	0.343	2,3,4,13	3	0.308	2,4,13

Numbers in bold indicate best correlation values for the selected combinations of abiotic variables. Numbers under Selection of Variables correspond to: electrical conductivity ‘EC’ (1), organic carbon ‘C’ (2), Olsen-phosphorus ‘P’ (3), NO_3_^−^ (4), NH_4_^+^ (5), moist (6), pH (7), elevation (9), fine sediments (10), region (11), moss in sample (12), and algae-cyanobacteria (13). Acronyms as following: Number of Variables (No.V), Correlation (Corr), Selection of Variables (Sel.V).

For rotifers, NO_3_^−^ was the best variable to explain composition (ρ = 0.075); and when combined, C, NO_3_^−^, elevation and region (ρ = 0.15) were the strongest variables. Rotifer abundance was better explained by P (ρ = 0.154) and by the combined effect of C, P and NO_3_^−^ (ρ = 0.205; Table 6). Individual Bioenv analyses based on presence/absence data were run for the bdelloid genera *Adineta* and *Philodina*. The highest correlation for *Adineta* corresponded to pH (ρ = 0.11) and NO_3_^−^ (ρ = 0.213); while for *Philodina*, corresponded to moist (ρ = 0.047) and slope (ρ = 0.037; Table 6). Bioenv results for tardigrade biotic parameters showed moist to be the best single variable to explain tardigrade composition and abundance (ρ = 0.107 and ρ = 0.141, respectively); and P, NO_3_^−^, moist and elevation to have the strongest combined effect (ρ = 0.179 for composition, and ρ = 0.141 for abundance; Table 7). We did not conduct separate analyses for tardigrade taxa, given that only few samples contained taxa other than Parachela (Echiniscoides in nine samples and Apochela in two samples). For ciliates, moist was the strongest variable to explain presence and abundance (ρ = 0.092 and ρ = 0.094, respectively). When considering a combination of variables it was seen that the highest correlation for ciliate presence involved NO_3_^−^, moist and slope (ρ = 0.129); and for ciliate abundance involved moist and slope (ρ = 0.135; Table 8). For mite presence, the highest correlation value (when one or more variables were considered) corresponded to NO_3_^−^ (ρ = 0.155), which was also the best value when correlated to mite abundance (ρ = 0.092); while the highest correlation value resulted for a combination of NO_3_^−^ together with moist, slope and elevation (ρ = 0.142; Table 9).

**Table 6:** Result from Bioenv analysis showing the strongest correlations for abiotic variables (when considered individually or in connection to others) that best match the biotic matrices for rotifer composition (all taxa combined, *Adineta* and *Philodina*), and abundance.

Rotifer composition									Rotifer abundance		
Rotifer taxa (all)			Adineta			Philodina					
No.V	Corr.(ρ)	Sel.V	No.V	Corr.(ρ)	Sel.V	No.V	Corr.(ρ)	Sel.V	No.V	Corr.(ρ)	Sel.V
1	**0.075**	4	1	**0.11**	7	1	**0.047**	6	1	**0.154**	3
1	0.062	3	1	0.075	4	1	0.037	8	1	0.134	1
1	0.039	5	4	**0.141**	4,6,7,11	1	0.035	14	1	0.121	5
4	**0.15**	2,4,9,11	3	**0.14**	3,4,7	4	**0.07**	6,8,9,14	1	0.107	2
3	**0.146**	2,4,9	4	0.138	3,4,7,11	4	0.069	8,9,12,14	4	**0.206**	1,2,3,4
4	0.146	2,4,9,15	3	0.138	4,6,7	3	**0.069**	8,9,14	4	0.206	2,3,4,7
4	0.146	2–4,9	2	**0.138**	4,7	3	0.069	6,9,14	3	**0.205**	2,3,4
4	0.142	1,2–4,9	4	0.137	3,4,6,7	4	0.068	3,6,9,14	4	0.205	2,3,4,5

Numbers in bold indicate best correlation values for the selected combinations of abiotic variables. Numbers under Selection of Variables correspond to: electrical conductivity ‘EC’ (1), organic carbon ‘C’ (2), Olsen-phosphorus ‘P’ (3), NO_3_^−^ (4), NH_4_^+^ (5), moist (6), pH (7), slope (8), elevation (9), region (11), moss in sample (12), soil from moss bed (14), and aspect (15). Acronyms as following: Number of Variables (No.V), Correlation (Corr), Selection of Variables (Sel.V).

**Table 7:** Result from Bioenv analysis showing the strongest correlations for abiotic variables (when considered individually or in connection to others) that best match the biotic matrices for tardigrade composition and abundance.

Tardigrade composition			Tardigrade abundance		
Number of Variables	Correlation (ρ)	Selection of Variables	Number of Variables	Correlation (ρ)	Selection of Variables
1	**0.107**	6	1	**0.141**	6
1	0.104	3	1	0.119	3
1	0.068	9	1	0.101	9
4	**0.179**	3,4,6,9	1	0.068	4
3	**0.176**	3,6,9	4	**0.238**	3,4,6,9
2	**0.156**	3,6	3	**0.222**	3,6,9
3	0.156	4,6,9	3	0.212	4,6,9
4	0.154	3,6,9,15	3	0.2	3,4,6
4	0.153	1,3,6,9	4	0.199	1,3,6,9
4	0.149	3,6,7,9	4	0.198	2,4,6,9

Numbers in bold indicate best correlation values for the selected combinations of abiotic variables. Numbers under Selection of Variables correspond to: electrical conductivity ‘EC’ (1), organic carbon ‘C’ (2), Olsen-phosphorus ‘P’ (3), NO_3_^−^ (4), Moist (6), pH (7), elevation (9), and aspect (15).

**Table 8:** Result from Bioenv analysis showing the strongest correlations for abiotic variables (when considered individually or in connection to others) that best match the biotic matrices for ciliate presence/absence and abundance.

Ciliate presence/absence			Ciliate abundance		
Number of Variables	Correlation (ρ)	Selection of Variables	Number of Variables	Correlation (ρ)	Selection of Variables
1	**0.092**	6	1	**0.094**	6
1	0.062	8	1	0.07	8
3	**0.129**	4,6,8	2	**0.135**	6,8
2	**0.128**	6,8	3	**0.134**	4,6,8
4	**0.107**	4,6,8,11	3	0.113	6,8,11
3	0.107	6,8,11	4	**0.113**	4,6,8,11
4	0.106	3,4,6,8	3	0.111	3,6,8
3	0.106	3,6,8	4	0.111	3,4,6,8
4	0.105	4,6,8,15	4	0.11	4,6,8,15

Numbers in bold indicate best correlation values for the selected combinations of abiotic variables. Numbers under Selection of Variables correspond to: Olsen-phosphorus ‘P’ (3), NO_3_^−^ (4), Moist (6), slope (8), region (11), and aspect (15).

**Table 9:** Result from Bioenv analysis showing the strongest correlations for abiotic variables (when considered individually or in connection to others) that best match the biotic matrices for mite presence/absence and abundance.

Mite presence/absence			Mite abundance		
Number of Variables	Correlation (ρ)	Selection of Variables	Number of Variables	Correlation (ρ)	Selection of Variables
1	**0.155**	4	1	**0.092**	4
1	0.058	10	1	0.06	10
1	0.05	7	1	0.057	9
2	**0.139**	4,7	1	0.043	6
4	**0.126**	4,8,10,13	4	**0.142**	4,6,8,9
4	0.124	4,5,7,10	4	0.14	4,5,6,9
4	0.123	4,7,8,13	4	0.136	4,5,6,10
4	0.122	4,7,10,13	4	0.135	4,6,7,10

Numbers in bold indicate best correlation values for the selected combinations of abiotic variables. Numbers under Selection of Variables correspond to: NO_3_^−^ (4), NH_4_^+^ (5), Moist (6), pH (7), slope (8), elevation (9), fine sediments (10), and algae-cyanobacteria (13).

Pearson correlation analysis (Table S3 in File S1) showed that microfauna abundance was positively correlated with C, NH_4_^+^, moisture, and fine sediments; and negatively correlated with pH and NO_3_^−^. Even when the three most common taxa (Rotifera, Tardigrada and Nematoda) were considered separately, positive correlations were observed for vegetation and C, while moisture was positively correlated with tardigrade, rotifer and ciliate abundance but not with nematodes (*Scottnema* showing a negative correlation). NO_3_^−^ was negatively correlated with mites, *Plectus* and *Eudorylaimus* presence, and with nematode abundance; while NH_4_^+^, P and C were all negatively correlated with *Eudorylaimus* and *Scottnema* presence. P and NH_4_^+^ were also found to have positive correlations with tardigrade and rotifer abundance (and ciliate abundance for NH_4_^+^); and EC showed correlations with *Scottnema* presence (negative) and ciliate presence and abundance (positive).

## Discussion

The present study is the most comprehensive to date in Antarctica correlating microfauna and environmental data across more than 2,000 km from East Antarctica (EA). It provides *i)* soil microfaunal composition and abundance; *ii)* soil geochemical data and vegetation content; and *iii)* correlations between soil geochemistry and other abiotic environmental variables with microfauna.

### Microfaunal distribution

There have been few soil microfaunal surveys for EA with most focusing on extremely restricted populations. Current knowledge on microfauna composition and abundance in EA is still incomplete, and in need of appropriate sampling. Considering previous research in other Antarctic regions, further sampling and molecular work is likely to reveal new species, resolve taxonomic problems and extend the known ranges of species. Studies for other Antarctic regions (Victoria Land) have revealed that nematodes were the most extensively distributed and abundant metazoan in soils [73]; but this is not the case for EA. In Dronning Maud Land, Sohlenius *et al.* [11] and Sohlenius & Boström [41] reported that the most commonly found taxon across samples were rotifers, followed by tardigrades and nematodes in similar proportions. Our results show that rotifers were also the most widespread group (Table 3e), followed by nematodes and then tardigrades (even though higher abundance was observed for tardigrades than nematodes), similar to previous studies in the VH [29].

#### Nematode occurrence and habitats

Nematode distribution in soil is affected by carbon content, moisture, and salinity [5], [74]; even though the environmental requirements vary depending on the species. We observed that soils with higher moisture content, C, P and NH_4_^+^ were inhabited predominantly by *Plectus*, while the opposite trend was observed for *Eudorylaimus* and *Scottnema*. *Scottnema* is reported to prefer dryer and saltier soils with lower organic matter than *Eudorylaimus* [5]. Based on Bioenv results (Table 5) we noticed that al-cy, NO_3_^−^, P and EC have an important contribution explaining nematode composition and abundance (no significant contribution was seen for moss samples). *Eudorylaimus* and *Scottnema* (Table 5) are driven by similar soil abiotic variables; with P, C, pH and NH_4_^+^ as strong drivers determining their presence. We observed *Scottnema* in soils with the lowest average EC (0.1 dS/m) and *Halomonhystera* in the highest (0.92 dS/m). Our findings support studies by Andrássy [43], [49] that reveal a tendency of *Halomonhystera* towards more saline environments. The distribution of *Eudorylaimus* from our study appears to correspond to their predatory habits on other nematode species [61], [75] whereby *Eudorylaimus* presence was always linked to potential prey (*Plectus* or *Scottnema*, but never in the presence of *Halomonhystera*) in a variety of soils with low-moderate C levels and for only 7% in samples with visible algae. Wall *et al.* [76] reported *Eudorylaimus* to be an algae-feeder and not an omnivore as previously recorded by others [15], [16], but our results did not show a correlation among *Eudorylaimus* presence and al-cy in sample (Table 5; though we did not account for microscopic algae).

*Scottnema* was present in dry and low-abundance populated soils, but with nematodes as the most abundant taxon, indicating the low carrying capacity for the species in the habitats targeted. Low densities for *Scottnema* observed here do not seem to correspond to other studies across Antarctica (e.g. [13], [16], [17], [61], [77] which report it as an abundant and widespread species. The lowest nematode density was seen for cf. Panagrolaimidae which has been reported for habitats rich in nitrogen, mostly linked to ornithogenic soils in the vicinity of bird colonies [23], [24], [78]. Of the five genera recorded for this study, *Plectus* was observed for the broadest geochemical ranges (N, C, P, EC and pH) indicating higher tolerance levels to environmental stresses. *Plectus* is a bacterial feeder, which potentially increases the range of habitats where it can be found. Nevertheless, denser populations were seen in presence of al-cy that could offer food as well as sheltered microhabitats. It is important to highlight that nematode presence was never as wide when considering NO_3_^−^ levels. We observed that samples with high NO_3_^−^ (23–1163 p.p.m.) only harbor tardigrades, ciliates and/or rotifers but no nematodes suggesting lower capabilities of the latter to adapt to NO_3_^−^ rich environments.

#### Rotifer occurrence and habitats

The presence of bdelloid rotifers in 87% of soil samples (Table 3e) reflects not only their broad distribution but also the high tolerance level of the group towards extreme conditions. Wide ranges in abiotic and geochemical parameters (EC, C, P, NO_3_^−^, NH_4_^+^ and pH) were observed for samples including bdelloids, suggesting that the effect of a single variable does not drive bdelloid composition and is more the result of a combination of abiotic factors (Table 6; Figs. 3, 5). Stronger contributions from single abiotic variables were observed by P, EC, NH_4_^+^ and C when considering rotifer abundance (Table 6). Our results also show a positive correlation between moisture and rotifer abundance (only seen for Pearson correlation analysis, Table S3 in File S1) as in other Antarctic regions [79]. Correlation of C with abundance was also reported by Sinclair & Sjursen [80] on Ross Island. We found 79% of total abundance occurred in samples with moss, algae or cyanobacteria. Soil pH seems to have an indirect role in determining abundance, given that three samples with higher bdelloid densities (LH-SP-04, MP-06 and SI-02) had low pH values (5.4–5.9) accounting for 45% of bdelloid abundance (Fig. 5). P and NH_4_^+^ also play an important role in bdelloid abundance; it was observed that a large proportion of bdelloids inhabit soils with moderate P content (69–123 mg/kg) representing 15% of total samples and accounting for almost half of rotifer abundance. Bdelloid numbers also seem to be indirectly affected by high NH_4_^+^ concentration in soils (98–373 p.p.m.) with contrasting results for the top 11 samples; four of those samples contributed 51% of the bdelloid abundance; but for three of those 11 samples no rotifers were observed.

#### Tardigrade occurrence and habitats

Tardigrades in the current study were mostly represented by the Order Parachela, a widely distributed Order reported elsewhere in Antarctica (e.g. [11], [12], [81], [82]). However, contrary to previous studies, we found no species of the genus *Pseudechiniscus* (Order Echiniscoidea), which has been reported as the most common tardigrade for the LH [83]. The Order Parachela was present in a variety of soil types, but mostly linked to soils with high levels of organic carbon and vegetation. In 98% of samples Parachela were found with bdelloid rotifers, suggesting similar habitat requirements, although rotifers were found across a greater range of soil properties (occurring with Parachela in 64% of cases). When looking at the Bioenv values for tardigrade abundance and composition we found soil moisture to be the strongest variable, followed by P, elevation and NO_3_^−^ (Table 7). Positive correlations between tardigrade abundance and moisture were also observed by Kennedy [19] and Freckman & Virginia [73] for Antarctic soils. However, the highest tardigrade densities in our study were in samples with contrasting soil moisture concentrations (77% and 1.2%; Fig. 6). It is likely that tardigrade moisture-abundance correlations were driven by three of the four high abundance samples with high moisture content (19–77%), accounting for more than 60% of tardigrade abundance. The strong correlation for vegetation and abundance (Table S3 in File S1; 97% of tardigrades in samples containing vegetation) was not reflected in the Bioenv values (Table 7). This could be explained as moisture driving vegetation growth and indirectly influencing tardigrade abundance.

The predatory and cosmopolitan species *Milnesium tardigradum* [29], [84] was only found in two highly diverse samples co-occurring with two other tardigrade Orders (Echiniscoidea and Parachela), nematodes (*Plectus*) and bdelloid rotifers. Their presence is likely to be linked to presence of other microfaunal taxa, probably reflecting their feeding habits, but this is only based on two samples. *Echiniscus* sp. was observed in 8% of samples with various taxa, but never in samples where *Scottnema* occurred, suggesting that most suitable habitats comprise moderate to high soil moisture concentration (9–67%) that is likely to be outside the optimum requirements for *Scottnema*.

#### Ciliates and habitats

Due to their minute size (∼70–100 µm in length) and oval shape, Ciliophora were not easily discernible. We restricted our study to live-mobile ciliates with visible cilia, and abundance and presence of ciliates/protozoans in our samples is likely to have been underestimated (ciliates present in 15% of samples). Other studies, based on Victoria Land, also reveal low ciliate frequency [81], [85] but high numbers of other protozoans (flagellates and amoeba). Ciliates were observed over a wide moisture range, but in our results most live-mobile ciliates (80% of samples) were present in high moisture soils (above 10%; Fig. 3) which might facilitate locomotion; also reported by Bamforth [86]. Bioenv values showed moist and slope to be the strongest abiotic variables influencing presence and abundance of ciliates in soils (Table 8). EC was reported to play a role in ciliate populations; studies from dry pond sediments [87] showed ciliate occurrence at high EC conditions (13–27 dS/m). In contrast, ciliates in our study were only found within EC ranges 0.04–4.4 dS/m, and only two of the 109 samples analysed were above 13 dS/m and neither with visible ciliates.

#### Mite occurrence and habitats

Mites were the largest invertebrates but the least frequent of all taxa in our samples. Previous studies for EA have revealed a general paucity of this taxon from most sampling locations due to possible micro-habitat preferences [88], [89]. Mites in Antarctica have been mostly linked to wet soils that support micro-algal growth (e.g. [9], [80], [89]); or in the vicinity of moss beds [22], [30]. Based on the Bioenv individual correlation values we observed NO_3_^−^ to be the strongest variable explaining mite presence and abundance (Table 9); no significant correlations were found for al-cy, moss, or proximity to moss beds with mite presence and abundance. Observations by Rounsevell [90] and Sinclair [24] who linked mite presence to food source (macroscopic algae), could not be supported with our findings, which show only 10% of samples to contain algae. In addition, most mites (56%) were present in soils with low to moderate moisture content (1.5–9.2%). Our data do not indicate a clear tendency for mites to favour wetter environments that sustain growth of algae; suggesting that other variables (besides moisture and algae) are influencing their presence. Convey [91] showed that temperature was the most obvious abiotic influence on micro-arthropod communities. The microclimate created by moss may provide a suitable habitat for mite survival, which are more susceptible to desiccation due to their size and permeable cuticle [92], [93]. The relative lack of samples with mites makes it difficult to tease out more complex associations, and abiotic variables other than vegetation, temperature and moisture are expected to be important. For example, we found that NO_3_^−^ together with FS, slope and elevation seems to play a relevant factor in determining mite abundance (Table 9).

### Correlating biotic and abiotic parameters

The Bioenv analyses linking abiotic variables with microfauna abundance and taxa composition (Tables 3–8) did not exceed correlation values of 0.206 when single variables were considered. Not surprisingly the effects were low since faunal ordination is not one-dimensional, and a single abiotic parameter does not provide a very successful match (e.g. [94]). Abiotic categories recorded during soil sampling and soil sieving did not play a major role compared to soil geochemical parameters. Single effects for the abiotic categories: region, aspect, slope, and particle size contributed poorly (or not at all) in determining interactions as seen in the PCA analysis (Fig. 3) and correlation values (Table 4–9). Salinity has been reported to influence diversity in Antarctic ecosystems. Magalhães *et al.* [9] showed a negative correlation between salt concentration and diversity; with salts increasing at higher elevations due to a longer exposure time of the terrain and more diluted in younger and active soils [7], [9]. We found in our study a substantially higher region (FM) than the other nine (Table 2), with the lowest average of total microfauna (Table 3c) and low to medium EC values (0.01–3.66 d S/m) which were used as a proxy for salinity (*viz*. [9]). In our studies, salinity was correlated with microfauna abundance and composition; but correlations were not evident for elevation (when single effect of variable was considered) and microfauna (Table 4). Other variables such as organic matter, soil moisture and microbial diversity have also shown to play a role in determining microfauna distribution and diversity in Antarctica (e.g. [31], [76]). In our study when organic C and soil moisture were correlated with microfauna abundance and composition, we noticed that microfauna composition was more strongly correlated to moisture, while microfauna abundance was more strongly correlated with C (Table 4). We did not account for microbial diversity.

Changes in soil geochemistry could be expected if seasonal variations are considered, in warmer periods we could expect higher accumulation of nutrients and C in lower sites as a result of greater meltwater (e.g. [13], [20]). Changes in microfauna distribution could also be affected as a result of water availability [90]. We found a strong correlation of biotic factors (taxa abundance and composition) with NO_3_^−^, P, EC, and pH; and we may expect biotic-abiotic correlations to be altered as a result of seasonal changes.

## Conclusions

We found that soil geochemical variables differed significantly among sites (Question 1) most likely as a result of variation in landscape formation/alteration, organic deposits from vegetation cover, ornithogenic inputs and shifting in nutrient accumulation due to melt-water runoff. Our study showed that abiotic variables are correlated with the composition of taxa (Question 2), with some taxa favouring *i)* close to neutral pH, drier and inorganic soils (*Scottnema*), *ii)* low NO_3_, neutral pH, low-medium organic soils (*Eudorylaimus*), *iii)* saltier and less vegetated soils (*Halomonhystera*), *iv)* soils higher in phosphorous, NH_4_^+^, C and moist (*Plectus*), *v)* more acidic soils without vegetation (*Echiniscus*), *vi)* acid-neutral soils high in moisture content *(Philodina)*, and *vii)* acidic soil (Adineta). Microfaunal abundance was significantly correlated with soil geochemistry (Question 3); we found that P, NO_3_^−^, EC and C are correlated with higher microfaunal densities of most common taxa (*Plectus murrayi*, *Adineta*, *Philodina* and Parachela); whereas in habitats with low pH, low moisture, low C, and high EC, the ‘specialists’ (also least abundant taxa) *Echiniscus, Scottnema, Eudorylaimus*, and *Halomonhystera* seem to do better. Our data indicate that region, slope and aspect did not play a major role in determining abundance. Our ability to address whether the occurrence of taxa is correlated with the presence of other taxa (Question 4) was confounded by determining if there is a biotic correlation among taxa, or if taxa co-occurring together are the result of similar micro-habitat requirements. It is most likely that any correlation (at least for non-predatory species) is the result of a connection between suitable soil geochemical conditions with soil productivity levels and microbial activity. For predatory species (*Milnesium*, and possibly *Eudorylaimus*), we could expect their distribution to reflect availability of prey, as they were always present with other taxa.

To understand soil microfaunal abundance, taxa composition and distribution in Antarctica it is important to determine their correlation with soil geochemistry and other environmental parameters. Where a population exists is likely to be determined by a suite of soil geochemical factors, with NO_3_^−^, P and salinity as the main drivers; and to a lesser extent by pH, C and soil moisture.

## Supporting Information

File S1 **Contains the following annexed supplementary tables.** **Table S1**, Location, abiotic parameters and meiofauna abundance for 109 samples from East Antarctica. **Table S2**, Measurements and de Man's ratios for Plectus murrayi and *Plectus murrayi* and *P. frigophilus* females from East Antarctica compared to other regions from various studies. **Table S3**, Pearson correlation matrix for 109 sites and the most relevant environmental and biotic variables.
(DOCX)
