# Supplementary material for: Distribution and Diversity of Soil Microfauna from East Antarctica: Assessing the Link between Biotic and Abiotic Factors
Source: PLoS One. 2014 Jan 31;9(1):e87529. doi: 10.1371/journal.pone.0087529 (PMC3909186; doi:10.1371/journal.pone.0087529)
Supplement: File S1 — Contains the following annexed supplementary tables. Table S1, Location, abiotic parameters and meiofauna abundance for 109 samples from East Antarctica. Table S2, Measurements and de Man's ratios for Plectus murrayi and Plectus murrayi and P. frigophilus females from East Antarctica compared to other regions from various studies. Table S3, Pearson correlation matrix for 109 sites and the most relevant environmental and biotic variables. (DOCX) [file pone.0087529.s001.docx]

**Supporting Information**

**File S1.** Annexed supplementary tables

Table S1.

Location, abiotic parameters and meiofauna abundance for 109 samples from East Antarctica

|  | **Elev (m)** | **Coordinates** | | **Cont** | **EC *dS/m*** | **C %** | **P *mg/kg*** | **NO_3_ *p.p.m*** | **NH_3_ *p.p.m*** | **Moist %** | **pH** | **Nem/ gdw** | **Tard/ gdw** | **Rot/ gdw** | **Cil/ gdw** | **Mit/ gdw** | **Abun/ gdw** |
| --- | --- | --- | --- | --- | --- | --- | --- | --- | --- | --- | --- | --- | --- | --- | --- | --- | --- |
| **Sample** |  | **South** | **East** |  |  |  |  |  |  |  |  |  |  |  |  |  |  |
| LH-SP-04 | 4 | -69.40 | 76.09 | al-cy | 0.88 | 9.88 | 69 | 3.4 | 372.6 | 77.05 | 5.9 | 2.79 | 672.1 | 934.2 | 0.00 | exu | 1609 |
| MP-06 | 70 | -68.85 | 77.94 | al-cy | 0.23 | 2.07 | 177.2 | 3.4 | 98.1 | 29.15 | 5.1 | 0.00 | 23.71 | 707.3 | 0.68 | 0.00 | 731.7 |
| SI-02 | 15 | -69.71 | 73.75 | moss | 0.14 | 2.69 | 43.9 | 10.8 | 20.1 | 1.22 | 5.4 | 0.00 | 140.8 | 483.9 | 0.00 | 0.00 | 624.7 |
| SI-03 | 15 | -69.71 | 73.75 | moss | 0.48 | 2.39 | 123.5 | 3.4 | 222 | 20.01 | 6.4 | 115.2 | 12.00 | 360.6 | 41.4 | 0.00 | 529.3 |
| VH-07 | 15 | -68.60 | 77.96 | al-cy | 0.25 | 0.09 | 8.9 | 6.8 | 15 | 18.96 | 8.2 | 0.00 | 75.83 | 397.2 | 0.00 | 0.00 | 473.0 |
| SI-01 | 20 | -69.71 | 73.75 | moss | 0.13 | 3.33 | 82 | 3.4 | 117 | 12.56 | 7.3 | 5.49 | 30.74 | 434.8 | 0.00 | 0.00 | 471.0 |
| LH-BP-10 | 68 | -69.39 | 76.38 | inorg | 0.02 | 0.28 | 28.2 | 3.4 | 5.1 | 6.42 | 5.2 | 0.00 | 0.00 | 303.9 | 0.00 | 0.03 | 304.0 |
| CS-08 | 30 | -66.28 | 110.52 | moss | 0.66 | 3.48 | 57.4 | 3.4 | 28.5 | 19.55 | 5.6 | 28.39 | 80.00 | 124.7 | 0.00 | 0.01 | 233.1 |
| L-Isl1-02 | 21 | -69.41 | 76.00 | moss | 0.13 | 1.46 | 113.9 | 3.4 | 14.1 | 25.85 | 5.2 | 0.65 | 49.49 | 156.7 | 0.00 | 0.00 | 206.9 |
| VH-10 | 6 | -68.50 | 78.08 | al-cy | 3.02 | 1.08 | 67.2 | 6.8 | 31.2 | 24.44 | 7.6 | 27.63 | 4.66 | 99.74 | 2.54 | 0.00 | 134.6 |
| MS-06 | 16 | -67.60 | 62.87 | lichen | 0.07 | 2.22 | 86.2 | 12.2 | 16.8 | 0.66 | 6.0 | 6.76 | 71.22 | 52.47 | 0.00 | 0.00 | 130.5 |
| VH-21 | 47 | -68.58 | 78.24 | al-cy | 3.5 | 5.48 | 1.9 | 4.1 | 7.8 | 58.39 | 6.7 | 0.00 | 16.15 | 22.30 | 85.6 | 0.00 | 124.0 |
| HI-10 | 32 | -68.83 | 77.68 | al-cy | 0.57 | 2.84 | 74.9 | 3.4 | 24.9 | 36.35 | 5.6 | 0.00 | 31.23 | 80.50 | 5.03 | 0.00 | 116.8 |
| MP-05 | 70 | -68.85 | 77.94 | moss | 0.06 | 0.79 | 38.4 | 3.4 | 4.8 | 12.06 | 6.5 | 14.93 | 11.35 | 89.02 | 0.10 | exu | 115.4 |
| LH-SP-06 | 10 | -69.40 | 76.09 | moss | 0.15 | 2.9 | 88.7 | 3.4 | 63.9 | 18.57 | 6.3 | 3.73 | 31.49 | 58.36 | 0.25 | 0.64 | 94.46 |
| HI-04 | 15 | -68.83 | 77.69 | moss | 0.29 | 0.27 | 53.3 | 3.4 | 10.5 | 5.45 | 5.2 | 0.76 | 3.81 | 66.17 | 0.00 | 0.00 | 70.74 |
| CS-13 | 34 | -66.28 | 110.53 | moss | 0.37 | 8.4 | 108.3 | 3.4 | 42.3 | 67.08 | 4.7 | 0.61 | 8.26 | 41.43 | 0.00 | 0.00 | 50.30 |
| CS-10 | 25 | -66.28 | 110.52 | moss | 0.45 | 6.14 | 60.7 | 3.4 | 35.1 | 19.4 | 5.4 | 16.50 | 10.12 | 21.84 | 0.00 | 0.12 | 48.59 |
| HI-05 | 15 | -68.83 | 77.70 | al-cy | 48.1 | 0.71 | 51.5 | 3.4 | 9.6 | 16.6 | 6.7 | 0.05 | 3.61 | 43.88 | 0.00 | 0.29 | 47.83 |
| HI-01 | 35 | -68.82 | 77.71 | al-cy | 0.94 | 0.77 | 60.5 | 41.5 | 43.5 | 1.88 | 6.0 | 0.00 | 0.00 | 44.76 | 1.69 | 0.00 | 46.45 |
| CS-06 | 31 | -66.28 | 110.52 | moss | 0.13 | 1.14 | 32.7 | 3.4 | 21.3 | 8.75 | 5.5 | 10.04 | 0.79 | 27.18 | 0.00 | 0.00 | 38.01 |
| VH-14 | 60 | -68.57 | 78.48 | moss | 0.41 | 3.64 | 122.1 | 6.8 | 57.3 | 1.14 | 6.1 | 0.16 | 0.00 | 28.12 | 0.00 | 0.00 | 28.28 |
| VH-09 | 6 | -68.50 | 78.08 | al-cy | 1.11 | 0.18 | 19.4 | 6.1 | 14.4 | 14.09 | 8.0 | 26.64 | 0.00 | 1.21 | 0.12 | 0.00 | 27.97 |
| MS-04 | 5 | -67.60 | 62.86 | al-cy | 0.25 | 0.38 | 10.8 | 3.4 | 6.6 | 3.79 | 6.7 | 0.00 | 17.88 | 9.27 | 0.00 | 0.00 | 27.15 |
| CS-05 | 30 | -66.28 | 110.52 | moss | 0.04 | 0.54 | 34.8 | 3.4 | 24.6 | 6.31 | 5.3 | 0.10 | 1.28 | 22.29 | 0.00 | 0.00 | 23.66 |
| VH-01 | 25 | -68.48 | 78.42 | inorg | 0.08 | 2.37 | 27.5 | 3.4 | 60.6 | 26.74 | 6.7 | 2.89 | 3.99 | 16.22 | 0.00 | 0.00 | 23.10 |
| CS-11 | 34 | -66.28 | 110.53 | inorg | 0.28 | 8.49 | 99.4 | 4.7 | 31.5 | 63.27 | 4.7 | 0.71 | 4.79 | 12.52 | 0.00 | 0.00 | 18.02 |
| L-Isl1-04 | 21 | -69.41 | 76.00 | moss | 0.03 | 2.4 | 27.1 | 3.4 | 11.4 | 24.18 | 4.8 | 1.27 | 0.95 | 14.77 | 0.00 | 0.11 | 17.09 |
| MS-03 | 24 | -67.60 | 62.87 | moss | 0.08 | 0.42 | 15.4 | 12.9 | 8.4 | 8.76 | 6.0 | 3.55 | 8.88 | 4.10 | 0.00 | 0.00 | 16.53 |
| L-Isl1-01 | 21 | -69.41 | 76.00 | inorg | 0.26 | 1.85 | 114.2 | 3.4 | 14.7 | 22.96 | 4.6 | 3.71 | 2.18 | 10.22 | 0.00 | 0.00 | 16.11 |
| HI-06 | 14 | -68.83 | 77.71 | al-cy | 0.81 | 0.97 | 4.2 | 4.1 | 27.9 | 28.62 | 6.9 | 13.11 | 0.00 | 0.10 | 0.00 | 0.00 | 13.21 |
| CS-12 | 34 | -66.28 | 110.53 | al-cy | 0.6 | 9.6 | 84.4 | 3.4 | 123.6 | 69.41 | 5.8 | 0.78 | 3.66 | 8.63 | 0.00 | 0.00 | 13.08 |
| MS-07 | 8 | -67.60 | 62.87 | inorg | 0.07 | 0.71 | 40.6 | 11 | 63.6 | 11.05 | 6.2 | 0.91 | 10.78 | 1.29 | 0.00 | 0.00 | 12.98 |
| LH-SP-07 | 41 | -69.40 | 76.09 | moss | 0.02 | 0.5 | 5.2 | 3.4 | 8.7 | 9.22 | 5.9 | 0.40 | 1.32 | 7.14 | 0.00 | 1.19 | 10.05 |
| HI-11 | 21 | -68.83 | 77.67 | al-cy | 0.08 | 0.52 | 184.3 | 3.4 | 77.1 | 11.51 | 6.1 | 0.00 | 3.98 | 4.88 | 0.00 | 0.00 | 8.86 |
| L-Isl2-02 | 27 | -69.37 | 76.14 | inorg | 0.07 | 0.67 | 14.7 | 3.4 | 36.9 | 14.59 | 5.8 | 0.28 | 3.37 | 4.68 | 0.00 | 0.00 | 8.34 |
| LH-SP-03 | 44 | -69.40 | 76.10 | moss | 0.03 | 1 | 15.6 | 3.4 | 28.8 | 0.28 | 5.8 | 0.01 | 2.93 | 4.72 | 0.00 | exu | 7.66 |
| MP-02 | 65 | -68.86 | 77.94 | lichen | 0.06 | 1.3 | 24 | 3.4 | 11.1 | 9.24 | 6.3 | 2.73 | 0.00 | 3.92 | 0.00 | 0.00 | 6.65 |
| LH-BP-12 | 1.6 | -69.38 | 76.38 | inorg | 0.09 | 0.58 | 7.3 | 3.4 | 5.1 | 20.17 | 5.8 | 0.05 | 5.61 | 0.41 | 0.00 | 0.00 | 6.08 |
| LH-SP-05 | 5 | -69.40 | 76.09 | moss | 0.4 | 6.76 | 65.6 | 3.4 | 13.8 | 42.83 | 4.7 | 0.07 | 2.31 | 3.31 | 0.14 | 0.00 | 5.82 |
| VH-18 | 23 | -68.60 | 78.29 | al-cy | 3.5 | 0.24 | 2 | 6.8 | 4.8 | 5.33 | 6.7 | 1.65 | 0.00 | 4.01 | 0.00 | 0.00 | 5.66 |
| CS-04 | 31 | -66.28 | 110.54 | inorg | 0.06 | 1.68 | 150 | 5.4 | 101.4 | 13.27 | 4.8 | 0.60 | 1.95 | 2.67 | 0.00 | 0.00 | 5.22 |
| VH-06 | 28 | -68.61 | 77.95 | inorg | 0.24 | 0.15 | 9.1 | 6.1 | 12.9 | 15.45 | 8.1 | 2.48 | 0.00 | 2.09 | 0.21 | 0.04 | 4.83 |
| LH-BP-02 | 54 | -69.38 | 76.38 | inorg | 0.03 | 0.1 | 8.4 | 3.4 | 18.6 | 11.86 | 6.4 | 1.70 | 0.07 | 2.76 | 0.00 | 0.00 | 4.53 |
| L-Isl2-04 | 27 | -69.37 | 76.14 | inorg | 0.44 | 0.95 | 4.7 | 3.4 | 4.8 | 18.45 | 4.3 | 0.17 | 0.78 | 2.94 | 0.00 | 0.00 | 3.90 |
| VH-02 | 17 | -68.64 | 78.30 | inorg | 0.1 | 0.22 | 11.6 | 3.4 | 9.1 | 10.75 | 6.1 | 3.80 | 0.00 | 0.00 | 0.00 | 0.00 | 3.80 |
| CS-01 | 28.4 | -66.28 | 110.53 | moss | 0.12 | 2.08 | 92.2 | 3.4 | 27.9 | 18.18 | 5.5 | 0.15 | 1.32 | 1.27 | 0.83 | 0.10 | 3.67 |
| LH-BP-03 | 16 | -69.39 | 76.39 | inorg | 0.04 | 0.19 | 7.8 | 3.4 | 7.2 | 11.84 | 6.3 | 0.16 | 2.48 | 1.02 | 0.00 | 0.00 | 3.66 |
| LH-SP-09 | 9 | -69.43 | 76.04 | inorg | 0.04 | 0.14 | 44.6 | 18.4 | 15.6 | 7.7 | 6.9 | 0.61 | 1.58 | 1.39 | 0.00 | 0.00 | 3.58 |
| FM-03 | 480 | -67.78 | 62.79 | inorg | 0.03 | 0 | 26 | 8.1 | 6.6 | 1.59 | 6.8 | 2.65 | 0.00 | 0.87 | 0.00 | 0.00 | 3.53 |
| MS-01 | 4 | -67.60 | 62.87 | al-cy | 0.05 | 0.45 | 49.3 | 16.9 | 8.4 | 2.9 | 6.3 | 0.14 | 0.10 | 3.05 | 0.00 | 0.00 | 3.30 |
| MP-01 | 70 | -68.86 | 77.94 | moss | 0.06 | 0.55 | 5.2 | 3.4 | 5.4 | 1.5 | 6.5 | 1.22 | 0.00 | 1.69 | 0.24 | exu | 3.15 |
| VH-15 | 47 | -68.57 | 78.48 | moss | 0.13 | 1.94 | 21 | 4.7 | 35.4 | 2.13 | 6.7 | 2.90 | 0.00 | 0.10 | 0.00 | 0.07 | 3.08 |
| MS-02 | 16 | -67.60 | 62.87 | moss | 0.09 | 3.66 | 79.1 | 4.7 | 12.9 | 33.46 | 5.5 | 1.89 | 0.63 | 0.32 | 0.02 | 0.00 | 2.86 |
| VH-11 | 6 | -68.50 | 78.08 | inorg | 0.38 | 0.18 | 12.1 | 7.5 | 6.9 | 5.85 | 6.1 | 0.15 | 0.02 | 2.06 | 0.00 | 0.55 | 2.78 |
| FM-02 | 490 | -67.78 | 62.79 | inorg | 0.11 | 0.05 | 22.3 | 12.2 | 7.5 | 6.53 | 7.6 | 0.06 | 0.06 | 2.23 | 0.09 | 0.00 | 2.44 |
| VH-20 | 25 | -68.60 | 78.24 | moss | 0.27 | 0.13 | 1.4 | 5.4 | 5.1 | 8.47 | 6.5 | 2.36 | 0.00 | 0.01 | 0.00 | 0.00 | 2.37 |
| HI-03 | 23 | -68.83 | 77.68 | inorg | 4.39 | 0.63 | 310.5 | 3.4 | 118.5 | 15.55 | 8.2 | 2.02 | 0.00 | 0.00 | 0.25 | 0.00 | 2.27 |
| CS-03 | 31 | -66.28 | 110.54 | moss | 0.06 | 3.13 | 171.2 | 3.4 | 57 | 21.23 | 5.1 | 0.05 | 1.12 | 1.02 | 0.00 | 0.00 | 2.18 |
| HI-15 | 14 | -68.83 | 77.71 | inorg | 0.14 | 0.29 | 10.1 | 3.4 | 13.5 | 15.1 | 6.7 | 0.28 | 0.05 | 1.32 | 0.33 | 0.00 | 1.98 |
| LH-BP-07 | 30 | -69.39 | 76.35 | inorg | 0.08 | 0.78 | 2.3 | 3.4 | 6.6 | 18.11 | 6.6 | 0.44 | 0.00 | 1.39 | 0.00 | 0.00 | 1.83 |
| VH-19 | 25 | -68.60 | 78.24 | moss | 0.05 | 0.17 | 5.1 | 3.4 | 12 | 5.78 | 6.0 | 0.82 | 0.50 | 0.48 | 0.00 | 0.00 | 1.79 |
| VH-05 | 15 | -68.51 | 78.51 | inorg | 0.16 | 0.01 | 41 | 14.2 | 17.4 | 0.91 | 7.8 | 0.16 | 0.01 | 1.56 | 0.00 | 0.00 | 1.74 |
| HI-02 | 31 | -68.82 | 77.70 | inorg | 0.1 | 0.45 | 97.8 | 3.4 | 6 | 1.81 | 7.2 | 0.00 | 0.00 | 1.63 | 0.00 | 0.00 | 1.63 |
| MP-03 | 45 | -68.86 | 77.93 | moss | 0.03 | 0.16 | 8.5 | 3.4 | 16.5 | 2.37 | 7.1 | 1.23 | 0.00 | 0.31 | 0.00 | 0.04 | 1.58 |
| VH-17 | 66 | -68.60 | 78.35 | moss | 0.02 | 0.48 | 8.7 | 3.4 | 4.5 | 2.25 | 6.9 | 1.37 | 0.00 | 0.19 | 0.00 | 0.00 | 1.57 |
| L-Isl2-03 | 27 | -69.37 | 76.14 | inorg | 0.22 | 0.52 | 8.5 | 3.4 | 4.8 | 16.3 | 5.8 | 0.73 | 0.22 | 0.55 | 0.00 | 0.00 | 1.49 |
| FM-06 | 460 | -67.77 | 62.82 | inorg | 0.01 | 0.01 | 18.7 | 8.8 | 15 | 1.29 | 5.7 | 0.01 | 0.00 | 1.46 | 0.00 | 0.00 | 1.47 |
| LH-BP-01 | 31 | -69.39 | 76.38 | al-cy | 0.08 | 0.2 | 32.3 | 3.4 | 6.9 | 10.72 | 5.4 | 0.03 | 0.01 | 1.28 | 0.00 | 0.03 | 1.36 |
| VH-08 | 50 | -68.63 | 78.41 | inorg | 0.26 | 0.17 | 11.3 | 3.4 | 33 | 8.51 | 7.9 | 0.27 | 0.00 | 0.86 | 0.00 | 0.11 | 1.25 |
| HI-13 | 12 | -68.83 | 77.73 | inorg | 0.04 | 0.07 | 6.3 | 3.4 | 7.2 | 15.18 | 7.5 | 0.28 | 0.00 | 0.73 | 0.19 | 0.00 | 1.20 |
| CS-09 | 25 | -66.28 | 110.52 | moss | 0.09 | 3.14 | 36.8 | 4.1 | 21.9 | 15.79 | 4.6 | 0.57 | 0.34 | 0.19 | 0.00 | 0.00 | 1.10 |
| L-Isl1-03 | 21 | -69.41 | 76.00 | moss | 0.03 | 0.28 | 40.9 | 3.4 | 5.7 | 1.54 | 5.8 | 0.41 | 0.08 | 0.55 | 0.00 | 0.00 | 1.04 |
| LH-BP-05 | 6 | -69.39 | 76.35 | inorg | 0.06 | 0.23 | 3.5 | 3.4 | 5.4 | 13.86 | 6.5 | 0.51 | 0.00 | 0.46 | 0.00 | 0.00 | 0.98 |
| VH-16 | 20 | -68.60 | 78.36 | inorg | 0.27 | 0.17 | 4.8 | 3.4 | 5.1 | 6.37 | 9.0 | 0.77 | 0.10 | 0.02 | 0.00 | 0.00 | 0.89 |
| VH-13 | 15 | -68.66 | 77.88 | inorg | 1.52 | 0.28 | 66.1 | 19 | 69.3 | 6.89 | 7.3 | 0.70 | 0.00 | 0.04 | 0.09 | 0.00 | 0.83 |
| LH-SP-02 | 40 | -69.40 | 76.10 | inorg | 0.02 | 0.06 | 13.9 | 3.4 | 8.1 | 7.06 | 6.2 | 0.15 | 0.09 | 0.47 | 0.00 | 0.00 | 0.71 |
| LH-SP-01 | 40 | -69.43 | 75.99 | inorg | 0.04 | 0.06 | 3.9 | 3.4 | 4.5 | 4.76 | 5.5 | 0.03 | 0.00 | 0.50 | 0.00 | 0.13 | 0.66 |
| L-Isl1-06 | 21 | -69.41 | 76.01 | moss | 0.02 | 0.6 | 9.5 | 3.4 | 5.1 | 1.08 | 5.9 | 0.00 | 0.00 | 0.65 | 0.00 | 0.00 | 0.65 |
| MP-07 | 80 | -68.85 | 77.94 | moss | 0.05 | 0.6 | 5.2 | 3.4 | 4.5 | 0.25 | 6.5 | 0.06 | 0.00 | 0.52 | 0.00 | exu | 0.58 |
| FM-05 | 460 | -67.77 | 62.82 | inorg | 0.04 | 0.01 | 12.5 | 23 | 12.9 | 4.77 | 5.8 | 0.00 | 0.00 | 0.57 | 0.00 | 0.00 | 0.57 |
| CS-14 | 4.2 | -66.28 | 110.54 | inorg | 0.16 | 1.29 | 107.4 | 3.4 | 9 | 16.43 | 4.5 | 0.00 | 0.00 | 0.51 | 0.00 | exu | 0.51 |
| LH-BP-06 | 40 | -69.39 | 76.35 | inorg | 0.03 | 0.21 | 5.3 | 3.4 | 23.7 | 12.06 | 8.0 | 0.02 | 0.05 | 0.41 | 0.00 | 0.00 | 0.48 |
| VH-22 | 47 | -68.58 | 78.24 | inorg | 0.13 | 0.01 | 2.5 | 4.1 | 5.4 | 9.42 | 7.5 | 0.00 | 0.03 | 0.43 | 0.00 | 0.00 | 0.46 |
| LH-BP-08 | 60 | -69.40 | 76.38 | inorg | 0.03 | 0.08 | 3.2 | 3.4 | 5.7 | 6.93 | 6.1 | 0.42 | 0.01 | 0.00 | 0.00 | 0.00 | 0.43 |
| LH-SP-08 | 59 | -69.40 | 76.12 | inorg | 0.03 | 0.11 | 4.3 | 3.4 | 6.3 | 12.67 | 6.2 | 0.13 | 0.00 | 0.15 | 0.00 | 0.06 | 0.33 |
| L-Isl1-05 | 21 | -69.41 | 76.01 | moss | 0.01 | 0.17 | 15.9 | 3.4 | 5.7 | 0.11 | 6.1 | 0.14 | 0.00 | 0.16 | 0.00 | 0.00 | 0.30 |
| CS-07 | 44 | -66.28 | 110.52 | moss | 0.23 | 3.6 | 169.3 | 3.4 | 18.9 | 27.73 | 5.7 | 0.00 | 0.00 | 0.00 | 0.00 | 0.29 | 0.29 |
| VH-03 | 5 | -68.51 | 78.51 | inorg | 0.03 | 0.1 | 9.9 | 3.4 | 30.9 | 5.23 | 7.6 | 0.27 | 0.00 | 0.00 | 0.00 | 0.00 | 0.27 |
| L-Isl1-07 | 21 | -69.41 | 76.01 | inorg | 0.04 | 0.01 | 4.2 | 3.4 | 4.5 | 1.78 | 6.5 | 0.12 | 0.03 | 0.04 | 0.00 | 0.04 | 0.23 |
| LH-BP-09 | 69 | -69.39 | 76.38 | inorg | 0.02 | 0.05 | 6.6 | 3.4 | 5.1 | 8.04 | 6.0 | 0.20 | 0.00 | 0.00 | 0.00 | 0.00 | 0.20 |
| LH-BP-04 | 60 | -69.39 | 76.39 | inorg | 0.06 | 0.06 | 5 | 3.4 | 6 | 7.62 | 6.9 | 0.03 | 0.00 | 0.15 | 0.00 | 0.00 | 0.18 |
| MP-04 | 44 | -68.85 | 77.94 | inorg | 0.03 | 0.01 | 5.2 | 3.4 | 5.1 | 13.71 | 6.1 | 0.05 | 0.05 | 0.08 | 0.00 | 0.00 | 0.17 |
| L-Isl2-01 | 27 | -69.37 | 76.14 | inorg | 0.22 | 0.11 | 4.6 | 3.4 | 4.2 | 1.26 | 6.7 | 0.13 | 0.00 | 0.00 | 0.00 | exu | 0.13 |
| LH-BP-14 | 46 | -69.39 | 76.33 | inorg | 0.02 | 0.11 | 3.2 | 3.4 | 6 | 1.14 | 7.2 | 0.08 | 0.00 | 0.05 | 0.00 | 0.00 | 0.13 |
| HI-14 | 36 | -68.83 | 77.74 | inorg | 0.02 | 0.01 | 3.6 | 3.4 | 5.4 | 0.44 | 7.8 | 0.12 | 0.00 | 0.00 | 0.00 | 0.00 | 0.12 |
| LH-BP-11 | 0 | -69.38 | 76.40 | inorg | 0.05 | 0.05 | 4.3 | 3.4 | 17.7 | 13.38 | 6.9 | 0.03 | 0.00 | 0.03 | 0.03 | 0.00 | 0.10 |
| FM-01 | 490 | -67.78 | 62.79 | inorg | 0.07 | 0.28 | 43.8 | 8.2 | 16.2 | 1.85 | 6.1 | 0.08 | 0.00 | 0.00 | 0.00 | 0.01 | 0.09 |
| VH-04 | 15 | -68.51 | 78.51 | inorg | 4.05 | 0.32 | 249.6 | 100.2 | 120 | 8.35 | 7.8 | 0.00 | 0.01 | 0.08 | 0.00 | 0.00 | 0.09 |
| LH-BP-13 | 25 | -69.39 | 76.32 | inorg | 0.06 | 0.08 | 4.5 | 3.4 | 4.8 | 11.71 | 7.2 | 0.00 | 0.00 | 0.08 | 0.00 | 0.00 | 0.08 |
| CS-02 | 28.4 | -66.28 | 110.53 | inorg | 0.03 | 0.17 | 146.1 | 3.4 | 31.5 | 7.64 | 5.3 | 0.00 | 0.00 | 0.04 | 0.00 | 0.01 | 0.05 |
| VH-12 | 4 | -68.66 | 77.87 | moss | 18.5 | 1.73 | 469 | 548.5 | 345 | 25.24 | 7.5 | 0.00 | 0.00 | 0.05 | 0.00 | 0.00 | 0.05 |
| HI-08 | 10 | -68.82 | 77.70 | inorg | 0.33 | 0.83 | 9.8 | 3.4 | 7.8 | 14.24 | 9.2 | 0.01 | 0.01 | 0.01 | 0.00 | 0.00 | 0.03 |
| FM-04 | 470 | -67.78 | 62.79 | inorg | 3.66 | 0.61 | 39.6 | 1163 | 12.3 | 6.87 | 6.5 | 0.00 | 0.01 | 0.02 | 0.00 | 0.00 | 0.03 |
| HI-09 | 33 | -68.83 | 77.69 | inorg | 0.1 | 0.03 | 9.2 | 3.4 | 7.2 | 10.9 | 8.6 | 0.01 | 0.00 | 0.00 | 0.00 | 0.00 | 0.01 |
| MS-05 | 16 | -67.60 | 62.86 | inorg | 0.96 | 0.15 | 63.7 | 17.7 | 9.9 | 1.87 | 6.1 | 0.00 | 0.00 | 0.00 | 0.00 | 0.00 | 0.00 |
| HI-16 | 25 | -68.83 | 77.68 | inorg | 1.66 | 0.57 | 217.3 | 142.8 | 117 | 10.43 | 5.0 | 0.00 | 0.00 | 0.00 | 0.00 | 0.00 | 0.00 |
| HI-12 | 14 | -68.83 | 77.72 | inorg | 0.08 | 0.01 | 18 | 3.4 | 7.2 | 0.81 | 7.2 | 0.00 | 0.00 | 0.00 | 0.00 | 0.00 | 0.00 |
| HI-07 | 15 | -68.83 | 77.70 | inorg | 0.37 | 0.37 | 242.1 | 40.1 | 153 | 7.12 | 6.3 | 0.00 | 0.00 | 0.00 | 0.00 | 0.00 | 0.00 |

Samples were sorted by total meiofauna abundance. Samples acronyms: Larsemann Hills-Broknes Peninsula (LH-BP), Larsemann Hills - Stornes Peninsula (LH-SP), Sansom Island (SI), Vestfold Hills (VH), Casey Station (CS), Hop Island (HI), Larsemann Islands (L-Isl), Mather Peninsula (MP), Mawson Station (MS), and Framnes Mountains (FM). Variables acronyms: Elevation (Elev), Vegetation content (Cont), algae-cyanobacteria (al-cy), soil with no visible photosynthetic material (inorg), electric conductivity (EC), organic carbon (C), Olsen-phosphorus (P), moisture (Moist), gdw (grams of dry weight of soil), nematodes (Nem), tardigrades (Tard), bdelloid rotifers (Rot), ciliates (Cil), mites (Mit), only mite exuviae (exu), and total abundance (Abun).

Table S2.

Measurements and de Man’s ratios for *Plectus murrayi* and *P. frigophilus* females from East Antarctica compared to other regions from various studies

| ***Plectus*** | **Region** | **N** | **Body length (µm)** | **Tail length (µm)** | **Width (µm)** | **Esoph. length (µm)** | **De Man’s ratios** | | |
| --- | --- | --- | --- | --- | --- | --- | --- | --- | --- |
|  |  |  |  |  |  |  |  |  |  |
|  |  |  |  |  |  |  | **‘a’** | **‘b’** | **‘c’** |
| *P*.cf. *murrayi* | **CS** | 5 | 800-920 | 85-110 | 28-38 | 165-225 | 22.1-28.9 | 3.8-4.8 | 7.4-9.4 |
| *P*.cf. *murrayi* | **VH** | 5 | 810-910 | 100-110 | 28-36 | 190-200 | 24.6-28.9 | 4.1-4.7 | 8.1-9.0 |
| *P*.cf. *murrayi* | **HI, MP** | 6 | 810-1080 | 80-110 | 33-44 | 170-250 | 22.3-28.4 | 4.3-4.8 | 7.8-10.8 |
| *P*.cf. *murrayi* | **BP, SP** | 7 | 800-1000 | 90-110 | 28-48 | 170-220 | 20-31.7 | 4.0-5.5 | 8.5-10.6 |
| *P*.cf. *murrayi* | **MS-FM** | 5 | 810-880 | 95-105 | 37-44 | 200-220 | 20.0-21.9 | 3.9-4.2 | 8.1-9.1 |
| *P. murrayi* | Gondwana (VL)^1^ | 16 | 817 ± 12 | 97 ± 2 | 31 ± 1 | 184 ± 1 | 26.5 ± 0.3 | 4.4 ± 0.1 | 8.5 ± 0.2 |
| *P. murrayi* | Dry Valley (VL) ^2^ | 10 | 750 - 840 | - | - | - | 24 - 28 | 3.8 - 4.4 | 7.6 - 8.8 |
| *P. murrayi* | Soya coast (EA)^3^ | 10 | 810-935 | 104-114 | 31-38 | 168-221 | 22.8-27.5 | 3.9-5.2 | 7.8-8.8 |
| *P. murrayi* | Marble Point (VL)^4^ | 25 | 600-820 | - | - | - | 15.2-24.8 | 4.7-6.0 | 6.5-9.1 |
| *P. murrayi* | Strand Moraines (VL)^4^ | 16 | 683-882 | - | - | - | 18.6-31.5 | 4.6-5.5 | 6.6-8.3 |
| *P. murrayi* | Bunger Hills (EA) ^5^ | 34 | 650-1000 | 98-128 | 31-52 | 151-200 | 16.2-23.7 | 3.7-5.2 | 6.1-8.6 |
| *P*.cf. *frigophilus* | **CS** | 1 | 1430 | 120 | 52 | 320 | 27.5 | 4.5 | 11.9 |
| *P*.cf. *frigophilus* | **BP, SP** | 4 | 1380-2050 | 120-140 | 50-55 | 280-420 | 26.5-37.3 | 4.1-4.9 | 9.9-14.6 |
| *P*.cf. *frigophilus* | **FM** | 1 | 1400 | 130 | 45 | 360 | 31.1 | 3.9 | 10.8 |
| *P. frigophilus* | McMurdo Sound^2^ | 10 | 1350-1720 | - | - | - | 24-33 | 4.9-5.4 | 9.9-11 |
| *P. frigophilus* | Edmonson Point (VL)^2^ | 5 | 1600-1820 | - | - | - | 23-24 | 4.7-5.0 | 11-12 |
| *P. frigophilus* | Soya coast (EA)^3^ | 4 | 1455-1700 | 137-161 | 50-62 | 310-357 | 26.1-29.1 | 4.5-4.8 | 9.3-11.4 |
| *P. frigophilus* | Bunger Hills (EA)^5^ | 25 | 1190-1580 | 120-160 | 38-56 | 290-350 | 25.5-33 | 4.0-4.8 | 9.2-10.9 |
| *P. frigophilus* | Marble Point (VL)^6^ | 10 | 1400-1990 | - | - | - | 22.2-32.5 | 4.4-5.2 | 9.7-12.3 |
| *P. frigophilus* | Strand Moraines (VL)^6^ | 10 | 1540-2060 | - | - | - | 25.7-30.0 | 4.5-5.1 | 10.5-13.5 |
| *P. frigophilus* | Obruchev Hills (EA)^7^ | 3 | 1360-1887 | - | - | - | 23-28 | 4.8-5.1 | 10.6-12.5 |

Measurements and ratios for *Plectus murrayi* and *P. frigophilus* female populations for the current study (in bold) compared to other regions across Antarctica. ^1^ shows data from Raymond [61] (‘mean ± SE’ are given for Gondwana populations); ^2^ indicates data from descriptions by Andrássy [27]; ^3^ data from species description by Kito et al. [50]; ^4^ indicates data taken from Yeates [95]; ^5^ shows measurements from EA by Yeates [96]; ^6^ indicates measurements and ratios from Timm [97]; and ^7^ shows the original measurements and ratios by Kirjanova [98]. Gaps indicate data not available. Acronyms: Casey Station (CS), Vestfold Hills (VH), Hop Island (HI), Mather Peninsula (MP), Larsemann Hills-Broknes Peninsula (BP), Larsemann Hills - Stornes Peninsula (SP), Mawson Station (MS), Framnes Mountains (FM), East Antarctica (EA), Victoria Land (VL). De Man’s ratios: ‘a’: total body length / maximum body diameter; ‘b’: total body length / total esophagus length; ‘c’: total body length / tail length.

**References**

27. Andrássy I (1998) Nematodes in the Sixth Continent. In: Peña Santiago R, editor. Journal of Nematode Morphlogy and Systematics. Jaén: Universidad de Jaén. pp. 107-186.

50. Kito K, Ohyama Y (2008) Rhabditid nematodes found from a rocky coast contaminated with treated waste water of Casey Station in East Antarctica, with a description of a new species of *Dolichorhabditis* Andrássy, 1983 (Nematoda: Rhabditidae). Zootaxa 1850: 43-52.

61. Raymond M (2010) Cold-temperature adaptation in nematodes from the Victoria Land Coast, Antarctica. Dunedin: University of Otago. 1-312 p.

95. Yeates GW (1970) Two Terrestrial Nematodes from the McMurdo Sound Region, Antarctica, with a Note on *Anaplectus arenicola* Killick, 1964. Journal of Helminthology 44: 27-34.

96. Yeates GW (1979) Terrestrial nematodes from the Bunger Hills and Gaussberg, Antarctica. New Zealand Journal of Zoology 6: 641-643.

97. Timm RW (1971) Antarctic Soil and Freshwater Nematodes from the McMurdo Sound Region. Proceedings of the Helminthological Society of Washington 38: 42-52.

98. Kirjanova ES (1958) Antarctic specimens of freshwater nematodes of the genus *Plectus bastian* (Nematoda, Plectidae). Information Bulletin of the Soviet Antarctic Expedition 3: 101-103.

Table S3.

Pearson correlation matrix for 109 sites and the most relevant environmental and biotic variables

|  | content | Elev | lnEC | lnC | lnP | lnNO3 | lnNH4 | lnMoist | lnpH | FS | Nem | Plectus | Eudor | Scott | Tard | Rot | Cil | Mit | lnNem_ab | lnTard_ab | lnRot_ab | lnCil_ab | lnAb_t |
| --- | --- | --- | --- | --- | --- | --- | --- | --- | --- | --- | --- | --- | --- | --- | --- | --- | --- | --- | --- | --- | --- | --- | --- |
| **content** | 1.00 | **-.194^*^** | 0.04 | **.545^**^** | **.244^*^** | -0.12 | 0.17 | -0.01 | **-.290^**^** | **.322^**^** | 0.06 | **.265^**^** | 0.04 | -0.17 | **.210^*^** | **.279^**^** | 0.08 | 0.18 | **.257^**^** | **.368^**^** | **.426^**^** | 0.13 | **.457^**^** |
| **Elev** | **-.194^*^** | 1.00 | -0.08 | **-.239^*^** | -0.02 | **.319^**^** | -0.11 | **-.211^*^** | 0.02 | **-.241^*^** | -0.02 | -0.07 | -0.09 | 0.11 | -0.15 | -0.04 | -0.02 | 0.00 | -0.13 | **-.206^*^** | -0.10 | -0.03 | -0.13 |
| **lnEC** | 0.04 | -0.08 | 1.00 | **.304^**^** | **.338^**^** | **.481^**^** | **.383^**^** | **.318^**^** | 0.15 | 0.07 | -0.17 | -0.11 | -0.17 | **-.258^**^** | 0.04 | -0.02 | **.231^*^** | -0.05 | -0.07 | 0.08 | 0.07 | .274^**^ | 0.08 |
| **lnC** | **.545^**^** | **-.239^*^** | **.304^**^** | 1.00 | **.548^**^** | -0.04 | **.531^**^** | **.477^**^** | **-.478^**^** | **.264^**^** | -0.01 | **.323^**^** | **-.222^*^** | **-.425^**^** | **.375^**^** | **.232^*^** | 0.15 | 0.08 | **.207^*^** | **.577^**^** | **.500^**^** | **.219^*^** | **.533^**^** |
| **lnP** | **.244^*^** | -0.02 | **.338^**^** | **.548^**^** | 1.00 | **.344^**^** | **.712^**^** | **.216^*^** | **-.334^**^** | **.214^*^** | **-.241^*^** | 0.02 | **-.471^**^** | **-.386^**^** | **.202^*^** | -0.04 | 0.13 | -0.01 | -0.08 | **.306^**^** | **.237^*^** | 0.13 | 0.18 |
| **lnNO3** | -0.12 | **.319^**^** | **.481^**^** | -0.04 | **.344^**^** | 1.00 | **.319^**^** | -0.09 | 0.11 | 0.10 | **-.350^**^** | **-.236^*^** | **-.189^*^** | -0.09 | -0.08 | -0.07 | -0.02 | **-.207^*^** | **-.315^**^** | -0.16 | -0.17 | -0.03 | **-.296^**^** |
| **lnNH4** | 0.17 | -0.11 | **.383^**^** | **.531^**^** | **.712^**^** | **.319^**^** | 1.00 | **.350^**^** | -0.09 | 0.16 | -0.09 | 0.07 | **-.321^**^** | **-.334^**^** | 0.16 | 0.00 | 0.18 | -0.07 | 0.06 | **.308^**^** | **.223^*^** | **.190^*^** | **.222^*^** |
| **lnMoist** | -0.01 | **-.211^*^** | **.318^**^** | **.477^**^** | **.216^*^** | -0.09 | **.350^**^** | 1.00 | **-.205^*^** | -0.09 | 0.06 | 0.19 | -0.17 | **-.334^**^** | **.369^**^** | 0.16 | **.248^**^** | -0.07 | 0.17 | **.419^**^** | **.262^**^** | **.264^**^** | **.313^**^** |
| **lnpH** | **-.290^**^** | 0.02 | 0.15 | **-.478^**^** | **-.334^**^** | 0.11 | -0.09 | **-.205^*^** | 1.00 | -0.12 | 0.13 | **-.265^**^** | 0.18 | **.276^**^** | **-.259^**^** | -0.13 | 0.14 | -0.14 | 0.02 | **-.341^**^** | **-.298^**^** | 0.12 | **-.234^*^** |
| **FS** | **.322^**^** | **-.241^*^** | 0.07 | **.264^**^** | **.214^*^** | 0.10 | 0.16 | -0.09 | -0.12 | 1.00 | -0.05 | 0.13 | 0.05 | -0.10 | 0.10 | 0.07 | 0.11 | 0.05 | 0.17 | 0.18 | 0.17 | 0.06 | **.228^*^** |
| **Nem** | 0.06 | -0.02 | -0.17 | -0.01 | **-.241^*^** | **-.350^**^** | -0.09 | 0.06 | 0.13 | -0.05 | 1.00 | **.633^**^** | **.354^**^** | **.327^**^** | 0.09 | 0.07 | 0.01 | 0.02 | **.795^**^** | 0.06 | 0.01 | -0.04 | 0.17 |
| **Plectus** | **.265^**^** | -0.07 | -0.11 | **.323^**^** | 0.02 | **-.236^*^** | 0.07 | 0.19 | **-.265^**^** | 0.13 | **.633^**^** | 1.00 | **.218^*^** | **-.236^*^** | **.265^**^** | 0.12 | -0.09 | 0.01 | **.576^**^** | **.310^**^** | **.230^*^** | -0.09 | **.326^**^** |
| **Eudor** | 0.04 | -0.09 | -0.17 | **-.222^*^** | **-.471^**^** | **-.189^*^** | **-.321^**^** | -0.17 | 0.18 | 0.05 | **.354^**^** | **.218^*^** | 1.00 | **.413^**^** | **-.230^*^** | -0.03 | -0.10 | **.212^*^** | **.323^**^** | **-.259^**^** | **-.215^*^** | -0.07 | -0.07 |
| **Scott** | -0.17 | 0.11 | **-.258^**^** | **-.425^**^** | **-.386^**^** | -0.09 | **-.334^**^** | **-.334^**^** | **.276^**^** | -0.10 | **.327^**^** | **-.236^*^** | **.413^**^** | 1.00 | **-.253^**^** | -0.06 | -0.07 | 0.11 | 0.18 | **-.314^**^** | **-.261^**^** | -0.09 | -0.19 |
| **Tard** | **.210^*^** | -0.15 | 0.04 | **.375^**^** | **.202^*^** | -0.08 | 0.16 | **.369^**^** | **-.259^**^** | 0.10 | 0.09 | **.265^**^** | **-.230^*^** | **-.253^**^** | 1.00 | **.386^**^** | 0.01 | -0.10 | 0.17 | **.861^**^** | **.522^**^** | 0.08 | **.477^**^** |
| **Rot** | **.279^**^** | -0.04 | -0.02 | **.232^*^** | -0.04 | -0.07 | 0.00 | 0.16 | -0.13 | 0.07 | 0.07 | 0.12 | -0.03 | -0.06 | **.386^**^** | 1.00 | 0.10 | 0.03 | 0.16 | **.363^**^** | **.712^**^** | 0.11 | **.517^**^** |
| **Cil** | 0.08 | -0.02 | **.231^*^** | 0.15 | 0.13 | -0.02 | 0.18 | **.248^**^** | 0.14 | 0.11 | 0.01 | -0.09 | -0.10 | -0.07 | 0.01 | 0.10 | 1.00 | -0.04 | 0.10 | 0.06 | 0.17 | **.918^**^** | **.224^*^** |
| **Acari** | 0.18 | 0.00 | -0.05 | 0.08 | -0.01 | **-.207^*^** | -0.07 | -0.07 | -0.14 | 0.05 | 0.02 | 0.01 | **.212^*^** | 0.11 | -0.10 | 0.03 | -0.04 | 1.00 | 0.07 | -0.05 | 0.07 | -0.02 | 0.08 |
| **lnNem_ab** | **.257^**^** | -0.13 | -0.07 | **.207^*^** | -0.08 | **-.315^**^** | 0.06 | 0.17 | 0.02 | 0.17 | **.795^**^** | **.576^**^** | **.323^**^** | 0.18 | 0.17 | 0.16 | 0.10 | 0.07 | 1.00 | **.224^*^** | **.216^*^** | 0.09 | **.415^**^** |
| **lnTard_ab** | **.368^**^** | **-.206^*^** | 0.08 | **.577^**^** | **.306^**^** | -0.16 | **.308^**^** | **.419^**^** | **-.341^**^** | 0.18 | 0.06 | **.310^**^** | **-.259^**^** | **-.314^**^** | **.861^**^** | **.363^**^** | 0.06 | -0.05 | **.224^*^** | 1.00 | **.701^**^** | 0.16 | **.702^**^** |
| **lnRot_ab** | **.426^**^** | -0.10 | 0.07 | **.500^**^** | **.237^*^** | -0.17 | **.223^*^** | **.262^**^** | **-.298^**^** | 0.17 | 0.01 | **.230^*^** | **-.215^*^** | **-.261^**^** | **.522^**^** | **.712^**^** | 0.17 | 0.07 | **.216^*^** | **.701^**^** | 1.00 | **.263^**^** | **.880^**^** |
| **lnCil_ab** | 0.13 | -0.03 | **.274^**^** | **.219^*^** | 0.13 | -0.03 | **.190^*^** | **.264^**^** | 0.12 | 0.06 | -0.04 | -0.09 | -0.07 | -0.09 | 0.08 | 0.11 | **.918^**^** | -0.02 | 0.09 | 0.16 | **.263^**^** | 1.00 | **.315^**^** |
| **lnAb_t** | **.457^**^** | -0.13 | 0.08 | **.533^**^** | 0.18 | **-.296^**^** | **.222^*^** | **.313^**^** | **-.234^*^** | **.228^*^** | 0.17 | **.326^**^** | -0.07 | -0.19 | **.477^**^** | **.517^**^** | **.224^*^** | 0.08 | **.415^**^** | **.702^**^** | **.880^**^** | **.315^**^** | 1.00 |

Variables preceded by ‘ln’ were subjected to log (x+0.1) transformation (after [68, 99]). Numbers in bold indicate correlation significant at 0.01 level (**) and 0.05 level (*). Abbreviation as following: vegetation (content), elevation (Elev), electric conductivity (EC), organic carbon (C), Olsen-phosphorus (P), moisture (Moist), fine sediment (FS), nematodes (Nem), *Eudorylaimus* (Eudor), *Scottnema* (Scott), tardigrades (Tard), rotifers (Rot), ciliates (Cil), mites (Mit), abundance (ab), total meiofauna (t). Headings: Plectus, Eudor, Scott, Tard, Rot, Cil and Mit show Pearson values for an original matrix of presence-absence. Type I error is an important aspect when conducting multiple comparison tests [100]. We do not correct probability values for our Pearson correlation since standard corrections like Holm’s sequential Bonferroni correction are found to be extremely conservative [101, 102]. Peres-Neto [100] suggests not using the Bonferroni test with a large number of comparisons given that the alpha value would be too small increasing the type II errors. Ellison & Gotelli [103] also suggest that adjusting the alpha value (significance level) is not recommended. A similar study (type of data/analyses) to ours that also uses Pearson correlations does not undertake any corrections for the reasons we cite above [104]. Here we do not apply a correction for multiple comparisons.

**References**

68. Knox MA, Hogg ID, Pilditch CA, Lörz A-N, Nodder SD (2012) Abundance and diversity of epibenthic amphipods (Crustacea) from contrasting bathyal habitats. Deep Sea Research Part I: Oceanographic Research Papers 62: 1-9.

99. Nielsen UN, Wall DH, Li G, Toro M, Adams BJ, et al. (2011) Nematode communities of Byers Peninsula, Livingston Island, maritime Antarctica. Antarctic Science 23: 349-357.

100. Peres-Neto PR (1999) How many statistical tests are too many? The problem of conducting multiple ecological inferences revisited. Marine Ecology Progress Series 176: 303-306.

101. Moran MD (2003) Arguments for rejecting the sequential Bonferroni in ecological studies. Oikos 100: 403-405.

102. Peres-Neto PR, Jackson DA, Somers KM (2003) Giving meaningful interpretation to ordination axes: assessing loading significance in principal component analysis. Ecology 84: 2347-2363.

103. Ellison GN, Gotelli N (2004) A primer of ecological statistics. Sinauer, Sunderland, Massachusetts, USA.

104. Cannone N., Wagner D, Hubberten, HW, Guglielmin M (2008) Biotic and abiotic factors influencing soil properties across a latitudinal gradient in Victoria Land, Antarctica. Geoderma 144: 50-65.
